# Early-pandemic wastewater surveillance of SARS-CoV-2 in Southern Nevada: Methodology, occurrence, and incidence/prevalence considerations

**DOI:** 10.1016/j.wroa.2020.100086

**Published:** 2020-12-31

**Authors:** Daniel Gerrity, Katerina Papp, Mitchell Stoker, Alan Sims, Wilbur Frehner

**Affiliations:** a Southern Nevada Water Authority, P.O. Box 99954, Las Vegas, NV, 89193, USA; b Department of Civil and Environmental Engineering and Construction, University of Nevada Las Vegas, 4505 S. Maryland Parkway, Box 454015, Las Vegas, NV, 89154, USA

**Keywords:** COVID-19, SARS-CoV-2, Wastewater surveillance, Sewage, Incidence, Prevalence

## Abstract

The World Health Organization (WHO) classified COVID-19 as a global pandemic, with the situation ultimately requiring unprecedented measures to mitigate the effects on public health and the global economy. Although SARS-CoV-2 (the virus responsible for COVID-19) is primarily respiratory in nature, multiple studies confirmed its genetic material could be detected in the feces of infected individuals, thereby highlighting sewage as a potential indicator of community incidence or prevalence. Numerous wastewater surveillance studies subsequently confirmed detection of SARS-CoV-2 RNA in wastewater and wastewater-associated solids/sludge. However, the methods employed in early studies vary widely so it is unclear whether differences in reported concentrations reflect true differences in epidemiological conditions, or are instead driven by methodological artifacts. The current study aimed to compare the performance of virus recovery and detection methods, detect and quantify SARS-CoV-2 genetic material in two Southern Nevada sewersheds from March–May 2020, and better understand the potential link between COVID-19 incidence/prevalence and wastewater concentrations of SARS-CoV-2 RNA. SARS-CoV-2 surrogate recovery (0.34%–55%) and equivalent sample volume (0.1 mL–1 L) differed between methods and target water matrices, ultimately impacting method sensitivity and reported concentrations. Composite sampling of influent and primary effluent resulted in a ∼10-fold increase in concentration relative to corresponding grab primary effluent samples, presumably highlighting diurnal variability in SARS-CoV-2 signal. Detection and quantification of four SARS-CoV-2 genetic markers (up to ∼10^6^ gene copies per liter), along with ratios of SARS-CoV-2 to pepper mild mottle virus (PMMoV), exhibited comparability with public health data for two sewersheds in an early phase of the pandemic. Finally, a wastewater model informed by fecal shedding rates highlighted the potential significance of *new* cases (i.e., incidence rather than prevalence) when interpreting wastewater surveillance data.

## Highlights

• 10X higher SARS-CoV-2 concentration in composite vs. early-morning grab samples.
• SARS-CoV-2 signal appears to exhibit diurnal variability.
• Coronavirus recovery ranged from 0.3 to 55% depending on sample processing approach.
• Secondary concentration to increase equivalent sample volume may not be warranted.
• SARS-CoV-2 RNA concentrations in wastewater fluctuated between 10^4^-10^6^ gc/L.

## Graphical abstract

**Figure:**
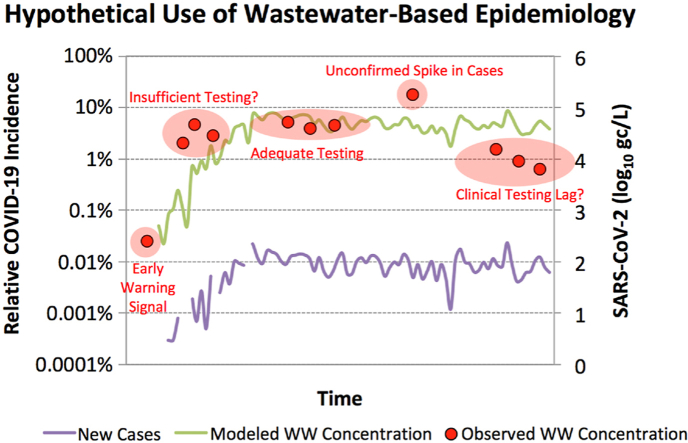

## 1.0 Introduction

Coronavirus disease 2019 (COVID-19) is caused by severe acute respiratory syndrome coronavirus 2 (SARS-CoV-2), otherwise known as the 2019 novel coronavirus (2019-nCoV). In late 2019, the first clusters of viral pneumonia of unknown origin had been identified in Wuhan, China (Lu et al., 2020), and by March 2020, the World Health Organization (WHO) had classified COVID-19 as a global pandemic (Bialek et al., 2020). Initial estimates of its case fatality rate (∼1–3%) were lower than SARS (11%), Middle East respiratory syndrome (MERS) (34%), and Ebola (25–90%) (Bialek et al., 2020; WHO, 2020a, 2020b, 2003). However, COVID-19’s propensity to spread before symptoms appeared in infected individuals—coupled with an overall asymptomatic ratio of >30% (Nishiura et al., 2020)—resulted in a relatively high reproduction number of 1.5–3.5 in the absence of mitigation measures (Eisenberg, 2020).

The severe morbidity and mortality outcomes ultimately led to extraordinary measures to mitigate effects on public health and the global economy, while also raising potential concerns for the water and wastewater industries. COVID-19 is primarily respiratory in nature, but early studies confirmed the presence of SARS-CoV-2 genetic material in the feces of infected individuals (Wölfel et al., 2020; Zang et al., 2020), possibly due to co-infection of cells within the gastrointestinal (GI) tract (Xiao et al., 2020). This alternative infection route was previously hypothesized for MERS (Zhou et al., 2017) and may explain why the genetic material of SARS-CoV-2 can be detected in feces even after it is no longer detected in oral and nasal swabs (Xiao et al., 2020). To date, isolation of *infectious* SARS-CoV-2 from fecal samples has been unsuccessful in multiple studies (Wölfel et al., 2020; Zang et al., 2020), although one study observed entry into Vero cells using SARS-CoV-2 isolated from a single patient (Zhang et al., 2020). Zang et al. (2020) hypothesized that although SARS-CoV-2 had the potential to infect cells within the GI tract, the virus appeared to be inactivated rapidly by colonic fluids, in contrast with enteric rotavirus. SARS-CoV-2 was not detected in urine in Wölfel et al. (2020), but infectious SARS-CoV-1 was detected in urine in the past (Xu et al., 2005).

Using knowledge gained from prior studies on SARS-CoV-2 surrogates (Bibby et al., 2017; Casanova et al., 2010; Gundy et al., 2009; Lytle and Sagripanti, 2005; Ye et al, 2016, 2018), a number of short communications provided initial assessments of SARS-CoV-2 risk for water and wastewater systems (Maal-Bared et al., 2020; Pecson et al., 2020; Wigginton and Boehm, 2020). But with respect to environmental applications, much of the scientific community focused on the application of wastewater surveillance or wastewater-based epidemiology (WBE) to characterize COVID-19 in communities throughout the world (Bivins et al., 2020). Wastewater surveillance involves monitoring chemical or microbiological targets to gain insight into the behaviors or characteristics of a community. This approach has been used to characterize opioid abuse (Gushgari et al., 2019), to facilitate polio eradication (Hovi et al., 2012), and as an ‘early warning’ signal for the spread of viral gastroenteritis (Hellmér et al., 2014). For SARS-CoV-2, this tool can potentially detect the initial occurrence or reemergence of COVID-19 in a local community (Medema et al., 2020), characterize trends in incidence or prevalence, and even complement case data to assess clinical testing coverage, in part because it captures both asymptomatic and symptomatic infections (WRF, 2020). As a ‘pooled’ sample, wastewater provides a broad representation of community health and can aid in characterizing viral strains circulating within a community with just a small number of samples (WRF, 2020).

Although much can be learned from existing literature on virus detection in environmental matrices (Haramoto et al., 2018; Ye et al., 2016), the rapid onset of COVID-19 presented a number of challenges for implementation of wastewater surveillance, particularly in identifying suitable methods for detection and quantification of SARS-CoV-2 (an enveloped RNA virus) in a complex, solids-rich matrix. Numerous studies have reported detection and/or quantification of SARS-CoV-2 genetic material in untreated wastewater (Ahmed et al., 2020; Gonzalez et al., 2020; Green et al., 2020; Medema et al., 2020; Miyani et al., 2020; Nemudryi et al., 2020; Sherchan et al., 2020; Wu et al., 2020b), treated wastewater (Wurtzer et al., 2020), and wastewater-associated solids (Peccia et al., 2020). However, the methods in these early studies vary widely, potentially confounding comparisons across studies. The objectives of the current study were to (1) characterize the performance of several sample collection, processing, and analysis methods; (2) attempt to detect and quantify SARS-CoV-2 genetic material in two Southern Nevada sewersheds and in wastewater-impacted surface water; and (3) better understand the potential link between clinically-confirmed COVID-19 case data and concentrations of SARS-CoV-2 RNA in wastewater. The knowledge gained during this early phase of the pandemic is valuable for continued efforts related to COVID-19 and for future implementation of wastewater surveillance to address other emerging public health challenges.

## 2.0 Methods

### 2.1 Sample collection

Water/wastewater samples were collected from the following five sites in Southern Nevada from early March 2020 to late May 2020: (1) a large, centralized wastewater treatment facility serving approximately 1 million people with a typical average daily flow of 105 million gallons per day (mgd) (Facility 1); (2) a smaller, satellite wastewater treatment facility serving approximately 60,000 people with a typical average daily flow of 5 mgd (Facility 2); (3) the Las Vegas Wash, a tributary of Lake Mead that consists primarily of treated effluent discharge from four major wastewater treatment facilities; (4) untreated surface water from Lake Mead; and (5) finished drinking water from three treatment facilities. For (3) through (5), weekly grab samples of 10–20 L were collected from those locations; details for (1) and (2) are provided below.

At Facility 1, routine monitoring included primary effluent grab samples collected between 10:00 a.m. and 11:00 a.m. each sampling day. The average hydraulic retention time (HRT) of the primary clarifier at this time is approximately 5 h, which suggests the grab samples corresponded with raw sewage that arrived at the facility between 5:00 a.m. and 6:00 a.m., when the flow rate is at a minimum (data shown later). For this sampling location, approximately 3% of the flow originates from solids dewatering. For routine monitoring, 100-mL samples were collected over the first two weeks of March (n = 4), and then 10-L samples were collected one to two times per week for the remainder of the study (n = 21). To compare grab versus composite and influent versus primary effluent, 10 composite influents and 4 composite primary effluents were also collected in tandem with the routine samples. All composites were 24-hr, flow-proportional, 1-L samples collected with a refrigerated autosampler. Several 10-L samples of secondary effluent (n = 4) and finished effluent (n = 2; activated sludge, secondary clarification, dual-media filtration, and UV disinfection) were collected to assess persistence of the SARS-CoV-2 genetic signal through wastewater treatment.

At Facility 2, routine monitoring included 24-hr, flow-proportional, composite influent samples collected one or two times per week starting in early April (n = 11). These samples were collected in 10-L volumes from a refrigerated autosampler.

### 2.2 Sample processing

The 10-L raw influent samples from Facility 2 were pre-filtered with 100-μm filter paper (Whatman grade 0965, GE Healthcare Bio-Sciences, PA, USA), and coarse solids were discarded. All other samples were processed without pre-filtration.

Primary concentration consisted of hollow fiber ultrafiltration (HFUF) (REXEED-25S, 30 kDa, Asahi Kasei Medical Co., Japan), centrifugal ultrafiltration (Centricon Plus-70, 30 kDa or 100 kDa, Millipore Sigma, Burlington, MA, USA), or polyethylene glycol (PEG) precipitation with 9% PEG 8000 (Promega Corporation, Madison, WI, USA) and 1 M sodium chloride (Thurston-Enriquez et al., 2003). For Centricon (initial volume up to 150 mL) and PEG (initial volume of 250 mL), samples were first centrifuged at 3500×*g* for 15–30 min at 10**°**C to pellet solids. PEG precipitation involved overnight mixing at 4**°**C, centrifugation at 3500×*g* for 60 min at 4**°**C, discarding of supernatant, and pellet resuspension in residual supernatant volume (∼0.5 mL). Preliminary experiments with PEG followed by chloroform-butanol extraction (1:1 v/v) indicated loss of SARS-CoV-2 signal so this extraction step was omitted from the study (see Table S5 for additional information). To maximize equivalent sample volume (ESV) for the Centricons, up to three successive centrifugations (3500×*g* for 20–30 min at 10**°**C) were performed, each time recording and discarding the filtrate volume and then adding additional sample volume to the device. More than three centrifugations sometimes resulted in apparent loss of Centricon filter column integrity, as indicated by unusually rapid filtrate passage and discoloration of the filtrate.

All samples with volumes ≥10 L were first processed by HFUF down to a volume of ∼100–200 mL, followed by centrifugation of the concentrate at 3500×*g* for 15–30 min at 10**°**C for solids pelleting. HFUF concentrates were either analyzed as-is or further processed with secondary concentration via Centricon ultrafilters (up to 150 mL) or PEG precipitation (40 mL). The resulting concentrates/pellets were immediately stored at −20 °C prior to nucleic acid extraction.

**Fig. 1:**
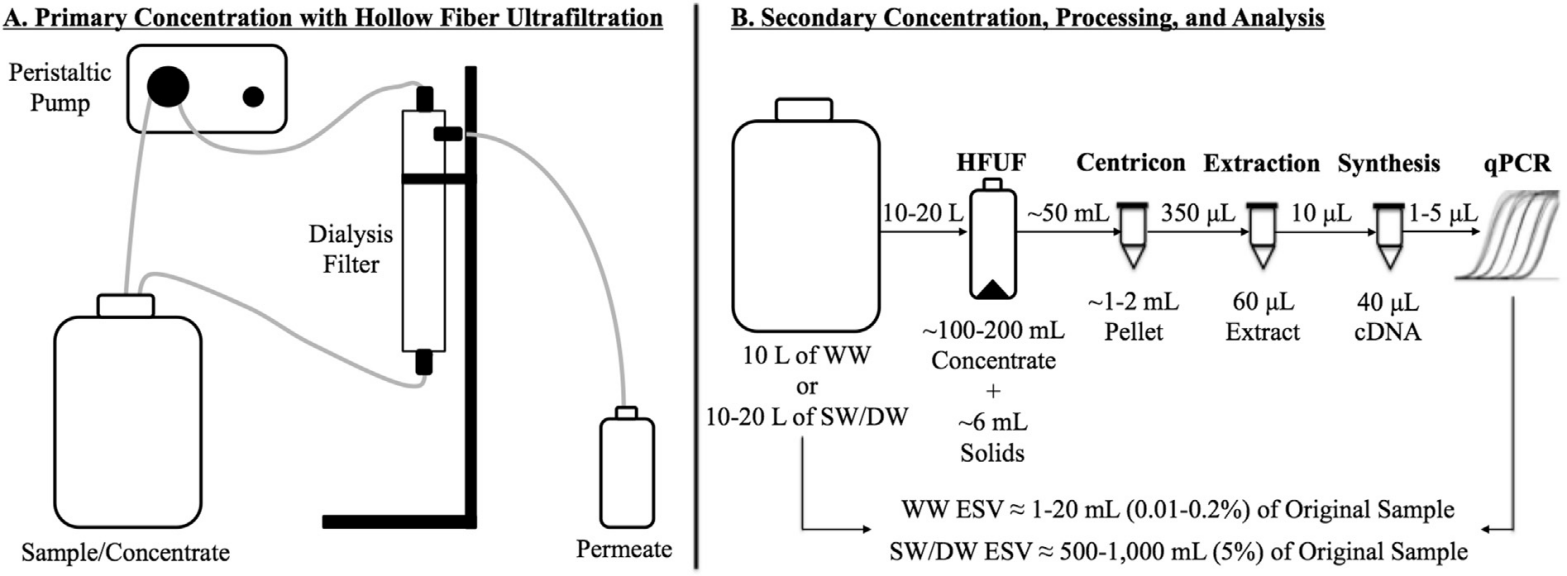
Schematic of (A) primary concentration with hollow fiber ultrafiltration (HFUF) and (B) secondary concentration with Centricon ultrafiltration, final sample processing, and analysis. This sample pipeline was used for weekly monitoring of wastewater (WW), surface water (SW), and drinking water (DW). This procedure resulted in equivalent sample volumes (ESVs) of 1–20 mL of wastewater, depending on the qPCR assay volume, and 500–1000 mL of surface water or drinking water.

With the exception of the first four samples collected at Facility 1, all routine monitoring samples were processed with the HFUF-Centricon approach (Fig. 1). For the first two weeks of March, 100-mL samples from Facility 1 were processed by centrifugation and Centricon ultrafiltration. This resulted in low overall ESVs of 0.2–1 mL, which prompted collection of larger sample volumes processed by HFUF-Centricon according to Papp et al. (2020) and Hill et al. (2007). Retrospectively, this may not have been the optimal approach, but since HFUF-Centricon had been used previously by our lab, it ensured greater consistency in trend analysis across the study.

The performance of each sample processing method was evaluated based on ESVs, recovery of spiked vaccine-strain bovine coronavirus (BCoV; Calf-Guard, Zoetis, Parsippany, NJ, USA), and recovery of native pepper mild mottle virus (PMMoV). Because the BCoV stock was acquired after the wastewater surveillance study began, recovery was evaluated in only a subset of the samples. Based on consistency in recovery efficiency, all SARS-CoV-2 concentrations were adjusted for average BCoV recovery (described later) from this sample subset. Additional details related to the BCoV recovery experiments are provided in Text S1.

### 2.3 Sample analysis

*Nucleic acid extraction and cDNA synthesis.* DNA and RNA in all liquid and solid samples were extracted in 350-μL volumes using the Purelink Viral RNA/DNA Mini Kit (Thermo Fisher Scientific, Waltham, MA) according to manufacturer’s instructions. Nucleic acids were quantified with a Qubit 3.0 Fluorometer and a dsDNA HS Assay kit or RNA HS assay kit (Thermo Fisher Scientific). Complementary DNA (cDNA) was directly synthetized from DNA/RNA extracts without dilution using the iScript™ Select cDNA Synthesis Kit (Bio-Rad Laboratories, Hercules, CA) or the Maxima First Strand cDNA Synthesis Kit (Thermo Fisher Scientific). All reactions were carried out in a Mastercycler gradient PCR system (Eppendorf, Hamburg, Germany) in 20–40 μL reaction volumes. Additional details for cDNA synthesis are available in Text S1.

*qPCR assays.* Wastewater samples were assayed for four SARS-CoV-2 gene targets using probe-based qPCR assays: N1 (CDC, 2020), N2 (CDC, 2020), E_Sarbeco (Corman et al., 2020), and *orf1a* (Lu et al., 2020); surface water and drinking water samples were assayed only for N1 and N2. All wastewater samples were also analyzed for PMMoV using a SYBR-based qPCR assay (Hamza et al., 2011). PMMoV has been identified as a useful indicator of fecal pollution because of its abundance in feces and sewage (Rosario et al., 2009). This RNA virus simultaneously serves as a valuable internal control for SARS-CoV-2 because its genetic material requires the same analysis pipeline (i.e., RNA extraction and cDNA synthesis). Spiked recovery samples were analyzed using a probe-based assay for BCoV (Decaro et al., 2008). Assay details are summarized in Table 1.

**Table 1:** Summary of the qPCR assays used in this study.

Assay	Target Virus	Primer name	Sequence (5’ → 3′)	Annealing Temp. (°C)	Reference
N1	SARS-CoV-2	2019-nCoV_N1–F	GACCCCAAAATCAGCGAAAT	55	CDC (2020)
		2019-nCoV_N1-R	TCTGGTTACTGCCAGTTGAATCTG		
		2019-nCoV_N1–P	FAM-ACCCCGCATTACGTTTGGTGGACC-BHQ1		
N2	SARS-CoV-2	2019-nCoV_N1–F	TTACAAACATTGGCCGCAAA	55	CDC (2020)
		2019-nCoV_N1-R	GCGCGACATTCCGAAGAA		
		2019-nCoV_N1–P	FAM-ACAATTTGCCCCCAGCGCTTCAG-BHQ1		
E_Sarbeco	SARS-CoV-2	E_Sarbeco_F	ACAGGTACGTTAATAGTTAATAGCGT	58	Corman et al. (2020)
		E_Sarbeco_R	ATATTGCAGCAGTACGCACACA		
		E_Sarbeco_P1	FAM-ACACTAGCCATCCTTACTGCGCTTCG-BBQ		
*orf1a*	SARS-CoV-2	orf1a_F	AGAAGATTGGTTAGATGATGATAGT	58	Lu et al. (2020)
		orf1a_R	TTCCATCTCTAATTGAGGTTGAACC		
		orf1a_Pb	FAM-TCCTCACTGCCGTCTTGTTG ACCA-BHQ1		
BCoV	Bovine Coronavirus	BCoV–F	CTGGAAGTTGGTGGAGTT	60	Decaro et al. (2008)
		BCoV-R	ATTATCGGCCTAACATACATC		
		BCoV–Pb	FAM-CCTTCATATCTATACACATCAAGTTGTT-BHQ1		
PMMoV (SYBR)	Pepper Mild Mottle Virus	PMMoV_F	GTGGCAGCAAAGGTAATGGT	55	Hamza et al. (2011)
		PMMoV_R	ATTTGCTTCGGTAGGCCTCT		

Primers, probes, and gBlock gene fragments for E_Sarbeco, *orf1a*, BCoV, and PMMoV were purchased from Integrated DNA Technologies (IDT, Skokie, IL, USA). The N1 and N2 reagents, including a plasmid-based positive control, were acquired as part of the 2019-nCoV RUO Kit from IDT, although N1 and N2 were quantified using a synthetic SARS-CoV-2 RNA standard from ATCC (VR-3276SD, ATCC, Manassas, VA, USA) (see Text S1). All assays were run on a CFX96 or CFX384 Touch™ Real-Time PCR Detection Systems (Bio-Rad Laboratories) using the conditions detailed in Text S1. Steps in determining limits of detection (LoDs), limits of quantification (LoQs), and ESVs are also described in Text S1, and corresponding data are summarized in Table 2.

**Table 2:** Calculated limits of detection (LoD) and limits of quantification (LoQ) for SARS-CoV-2 routine monitoring. Concentrations are reported per L of original sample after accounting for equivalent sample volume (ESV) and recovery. The LoDs and LoQs have ranges due to the differences in ESV for each sample. Also, the LoDs and LoQs are higher for E and *orf1a* due to their lower ESVs.

Parameter	Sample	LoD^1^	N1 LoQ^1^	N2 LoQ^1^	LoD^2^	E LoQ^2^	*orf1a* LoQ^2^
**qPCR Reaction**	N/A	2	10	20	1	5	5
**(gc)**							
**Centricon**^**3**^	WW	(3.7 ± 0.5) × 10^3^	(1.8 ± 0.3) × 10^4^	(3.7 ± 0.5) × 10^4^	(9.2 ± 1.3) × 10^3^	(4.6 ± 0.7) × 10^4^	(4.6 ± 0.7) × 10^4^
**(gc/L)**							
**HFUF-Centricon**^**4**^	WW	(8.5 ± 3.1) × 10^3^	(4.2 ± 1.5) × 10^4^	(8.5 ± 3.1) × 10^4^	(2.1 ± 0.8) × 10^4^	(1.1 ± 0.4) × 10^5^	(1.1 ± 0.3) × 10^5^
**(gc/L)**							
**HFUF-Centricon**^**4**^	SW/DW	(1.4 ± 0.7) × 10^2^	(7.1 ± 3.4) × 10^2^	(1.4 ± 0.7) × 10^3^	N/A	N/A	N/A
**(gc/L)**							

LoD = limit of detection; LoQ = limit of quantification; HFUF = hollow fiber ultrafiltration; gc = gene copy; WW = wastewater; SW = source water; DW = drinking water; ^1^qPCR reaction with 5 μL of cDNA template; ^2^qPCR reaction with 1 μL of cDNA template; ^3^Sample concentrations adjusted for 55% recovery; ^4^Sample concentrations adjusted for 2.1% recovery.

Inhibition was assessed by two different methods. First, a known concentration of the IDT N1/N2 plasmid—used as an internal positive control (IPC)—was spiked into several cDNA samples generated from HFUF-Centricon concentrates. Inhibition was assumed to be absent or indiscernible based on consistency in cycle of quantification (Cq) between spiked cDNA and spiked blanks (i.e., Cq = ±1.0). Second, a separate IPC (QuantiFast Pathogen PCR + IC kit, Qiagen, Hilden, Germany) was spiked according to manufacturer’s instructions to assess inhibition in another subset of wastewater-derived cDNA samples. Again, inhibition was assumed to be absent or indiscernible based on the Cq criterion.

## 3.0 Results and discussion

The following samples were non-detect for SARS-CoV-2 RNA: secondary (n = 4) and finished (n = 2) wastewater effluents from Facility 1 (no treated wastewater samples were collected from Facility 2), blended wastewater effluent in the Las Vegas Wash (n = 11), untreated surface water from Lake Mead (n = 11), and finished drinking water (n = 33). Because these samples were all non-detect, the remainder of this study focuses on wastewater influent and primary effluent.

### 3.1 Method comparisons

Because of the rapid onset of COVID-19 and time-sensitive nature of this research, it was not possible to identify optimized sample collection, processing, and analysis methods prior to the study. Therefore, relatively consistent methods were employed for routine monitoring, but those were supplemented with additional experiments and modeling to characterize the impacts of methodological considerations on the SARS-CoV-2 genetic signal in wastewater.

The SARS-CoV-2 genetic signal can potentially be affected by sample location, sample type, and time of collection. For example, the signal may change depending on whether the sample is collected upstream in a sewershed, in the influent to a wastewater treatment facility, or in the primary clarifier effluent, primarily due to differing compositions, dilutions, and dispersion effects. The signal may also depend on whether the sample is a grab versus composite, the type of composite (e.g., time-proportional vs. flow-proportional), and the time of day when it is collected (Ort et al., 2010b). Particularly early in the outbreak, SARS-CoV-2 might be considered a rare constituent, in contrast with more ubiquitous indicators of fecal contamination such as PMMoV, so its detection/presence at any given time may be more susceptible to hydraulic effects. Ort et al. (2010a) showed that some rare constituents exhibit intermittent concentration peaks that might be missed with mistimed grab samples.

Text S2 summarizes the potential effects of hydraulics on SARS-CoV-2 concentrations with several hypothetical scenarios. As shown in Fig. S1 (non-ideal reactor with dispersion), primary clarifiers have the potential to disperse the signal from a rare constituent over several hours. This increases the probability of capturing the signal but potentially dilutes the signal strength. To compensate for the potential dilution effect, larger sample volumes (e.g., 10 L) were initially assumed to be necessary. It was not possible to obtain composite sample volumes greater than 1 L at Facility 1 so 10-L grab samples of primary effluent were initially selected for routine monitoring in this study.

To test the effects of sample location, sample type, and potentially sample time, the genetic signal from SARS-CoV-2 was compared in composite influent (Centricon only), composite primary effluent (Centricon only), and grab primary effluent samples from Facility 1 (HFUF-Centricon). For both SARS-CoV-2 and PMMoV, the concentrations adjusted for method-specific recovery were consistently higher in both composite samples (Table S3), which was also observed in Curtis et al. (2020). SARS-CoV-2 concentrations were 1 order of magnitude higher on average, and PMMoV concentrations were 1–2 orders of magnitude higher. As shown in Fig. S1, dispersion might cause primary effluent concentrations to be lower than raw influent samples for a rare constituent, but with the exception of one day for PMMoV, concentrations of SARS-CoV-2 and PMMoV were similar between corresponding composite influents and composite primary effluents. Therefore, the consistently higher signals in the composite samples might be attributable to time differences. The grab primary effluent samples corresponded with the minimum flow for Facility 1 so loadings of SARS-CoV-2 and PMMoV might increase later in the day. Additional testing would be needed to confirm this diurnal variation.

With respect to sample processing, numerous approaches were evaluated throughout the duration of the study. The methods were evaluated based on equivalent sample volume and recovery of spiked BCoV and native PMMoV, for which data are summarized in Table 3. It was initially assumed that secondary concentration methods would be needed to maximize ESV for SARS-CoV-2 detection, hence the selection of HFUF-Centricon for routine monitoring of 10-L samples. Hill et al. (2007) previously demonstrated >70% recovery of MS2 and phiX174 using this combined approach. Although secondary concentration was able to increase ESVs (e.g., 0.55 ± 0.05 mL for HFUF vs. 11 ± 2.8 mL for HFUF-Centricon), secondary concentration resulted in a notable decrease in BCoV recovery (e.g., 54 ± 11% for HFUF vs. 2.1 ± 0.87% for HFUF-Centricon). Furthermore, spiking BCoV at different stages of sample processing (see Text S1) confirmed that performance deteriorated during secondary concentration. For example, primary concentration with Centricon resulted in a high yet variable BCoV recovery (55 ± 38%), similar to the MS2 recovery observed in Medema et al. (2020). However, when isolating secondary concentration, BCoV recovery by Centricon dropped to 9.4 ± 9.5%. A similar decrease in performance was observed for PEG when used for secondary concentration following HFUF, with the overall HFUF-PEG method achieving only 0.34% recovery of BCoV. In contrast, using PEG for primary concentration resulted in higher recoveries of 11 ± 8.4%. In a small number of split samples, direct extraction of the HFUF concentrate appeared to be slightly more sensitive than the overall HFUF-Centricon approach. HFUF alone achieved a greater number of positive qPCR assays/reactions in 8 of 10 split samples, although concentrations corrected for ESV and recovery were nearly identical between the two methods (<1.5-fold difference for SARS-CoV-2 and PMMoV). For all methods, PMMoV recovery was ∼1 order of magnitude lower than BCoV recovery.

**Table 3:** Summary of equivalent sample volumes (ESVs) and virus recoveries as a function of sample concentration method and target matrix. Sample concentration and analysis focused on liquid-phase viruses, except where solids are specified. The HFUF-Centricon method was applied to wastewater (10-L samples) and source/drinking water (10–20-L samples). All other methods were tested only with wastewater. ESVs indicate the volume of original sample reflected in each assay after accounting for sample concentration, sample processing, and molecular analyses.

Primary Concentration	Secondary Concentration	Average ESV^1^ (mL)	Average ESV^2^ (mL)	BCoV Recovery (n = 3–4)	PMMoV Recovery (n = 2–4)
HFUF	None	0.55 ± 0.05	0.11 ± 0.01	54% ± 11%	4.0% ± 2.2%
HFUF	Centrifuge (Solids)^3^	∼12	∼2.4	0.18% ± 0.09%	0.02% ± 0.01%
Centricon	None	1.1 ± 0.78	0.22 ± 0.16	55% ± 38%	1.3% ± 0.16%
PEG	None	3.6 ± 0.07	0.72 ± 0.01	11% ± 8.4%	0.28% ± 0.10%
HFUF	PEG	53 ± 18	11 ± 3.6	0.34%^5^	0.01%^5^
HFUF	Centricon	11 ± 2.8	2.2 ± 0.57	2.1% ± 0.87%	0.12% ± 0.05%
HFUF^4^	Centricon	500-1000	N/A	N/A	N/A

HFUF = hollow fiber ultrafiltration; PEG = polyethylene glycol; ESV = equivalent sample volume; BCoV = bovine coronavirus; PMMoV = pepper mild mottle virus; ^1^ESV for N1 and N2 assays (5 μL per reaction); ^2^ESV for E_Sarbeco, *orf1a*, BCoV, and PMMoV (1 μL per reaction); ^3^Recovery for solids may actually reflect low recovery and/or low virus partitioning to solids; ^4^Data for source/drinking water samples (only N1 and N2 assays and no recovery experiments performed in these matrices); ^5^Recovery evaluated in a single sample.

Considering the higher observed concentrations in composite samples with reduced volume, the lack of a clear benefit of secondary concentration, and the costs associated with additional processing, primary concentration with HFUF (current study), PEG precipitation (Wu et al., 2020b), Centricon ultrafilters (Medema et al., 2020), or electronegative filtration (Gonzalez et al., 2020) should be adequate for wastewater surveillance of SARS-CoV-2. Other viable options appear to be ultracentrifugation (Green et al., 2020; Wurtzer et al., 2020) or even direct extraction from sludge (Peccia et al., 2020). In the current study, BCoV concentrations were low for extracted solids from spiked samples (Table 3), although solids partitioning in the recovery experiments may not have accurately mimicked partitioning in actual samples (samples were rocked for 10 min after spiking with BCoV). Ye et al. (2016) estimated ∼15% adsorption of enveloped viruses to wastewater solids under equilibrium conditions. During routine monitoring, only 26% and 13% of extracted solid samples from Facility 1 and Facility 2, respectively, were positive for one or more SARS-CoV-2 assays (maximum of 2 positive assays and 2 positive reactions for a single sample). Peccia et al. (2020) employed a nucleic acid extraction kit specifically designed for solid samples, which may have improved recovery from sludge and SARS-CoV-2 detection in that study.

### 3.2 COVID-19 case data and wastewater occurrence of SARS-CoV-2

**Fig. 2:**
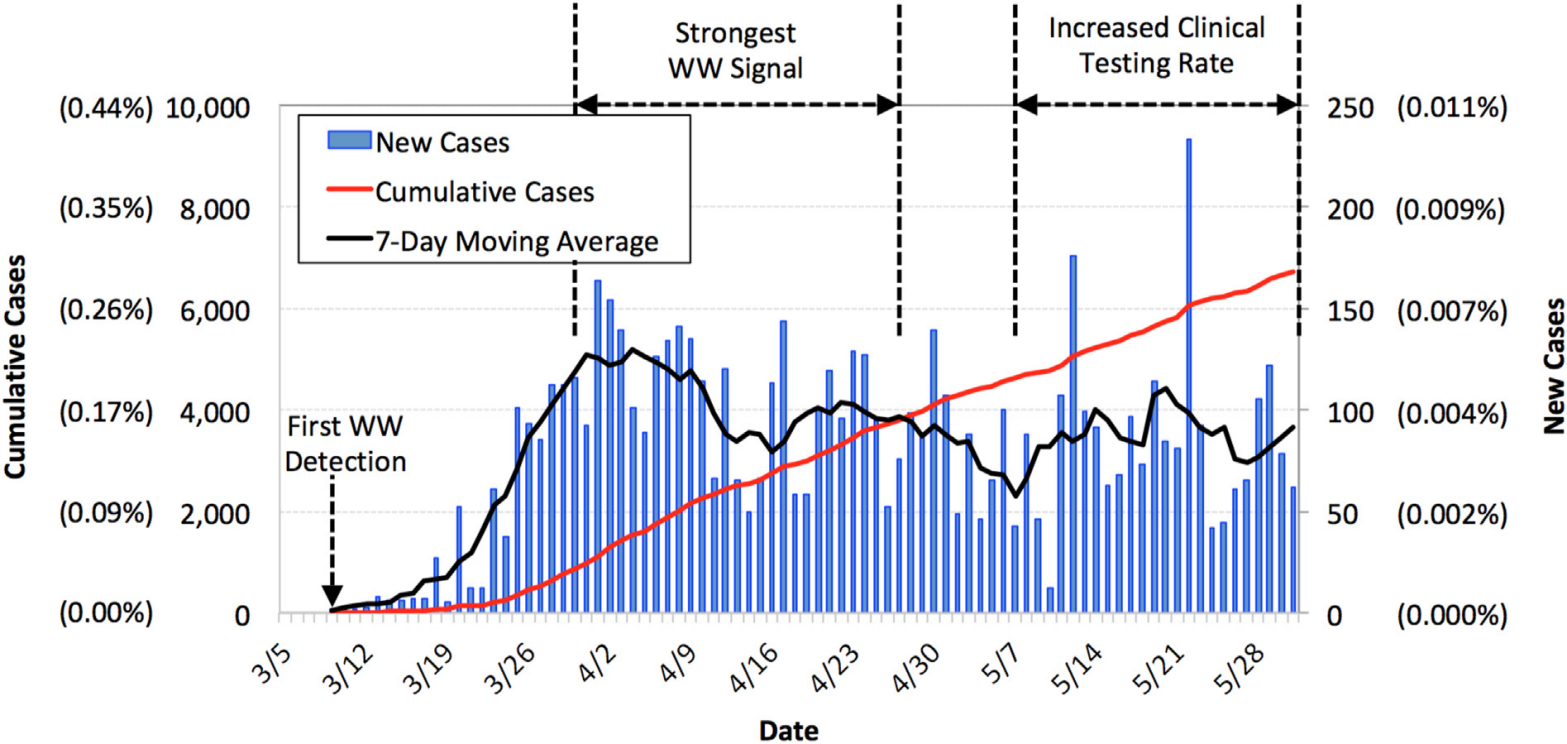
Clinically confirmed COVID-19 case data for Southern Nevada (SNHD, 2020). New cases were assigned based on when data were reported to the Southern Nevada Health District (SNHD) and not based on symptom onset. Numbers in parentheses indicate case data relative to an overall population of approximately 2.3 million people. A statewide closure of non-essential businesses was ordered on March 17th, phase 1 reopening began on May 9th, and phase 2 reopening began on May 29th.

Fig. 2 summarizes the clinical case data for Southern Nevada from March 9th (first confirmed cases) through May 31st (SNHD, 2020). The raw data from the Southern Nevada Health District (SNHD) correspond with the date of positive test confirmation rather than onset of symptoms, so Fig. 2 may include a time lag. Nevertheless, the data provide some indication of when COVID-19 initially spiked and then peaked in the community—at which point just over 0.007% of the population was confirmed positive each day. The number of new cases generally waned after the first week of April but then increased in early May when there was an increase in testing rate. The constant decrease in hospitalizations and deaths through the end of May (Fig. S2), coupled with a higher frequency of non-detects in wastewater (described later), suggest that the increase in new cases in early May was largely a function of increased testing. Following the peak, the 7-day moving average fluctuated between 57 and 111 new cases per day—or 0.002–0.005% of the population. By the end of May, there were 6719 confirmed cases of COVID-19 (0.29% relative prevalence), 1565 related hospitalizations (23% of confirmed cases), and 343 related deaths (5% of confirmed cases) in Southern Nevada.

**Fig. 3:**
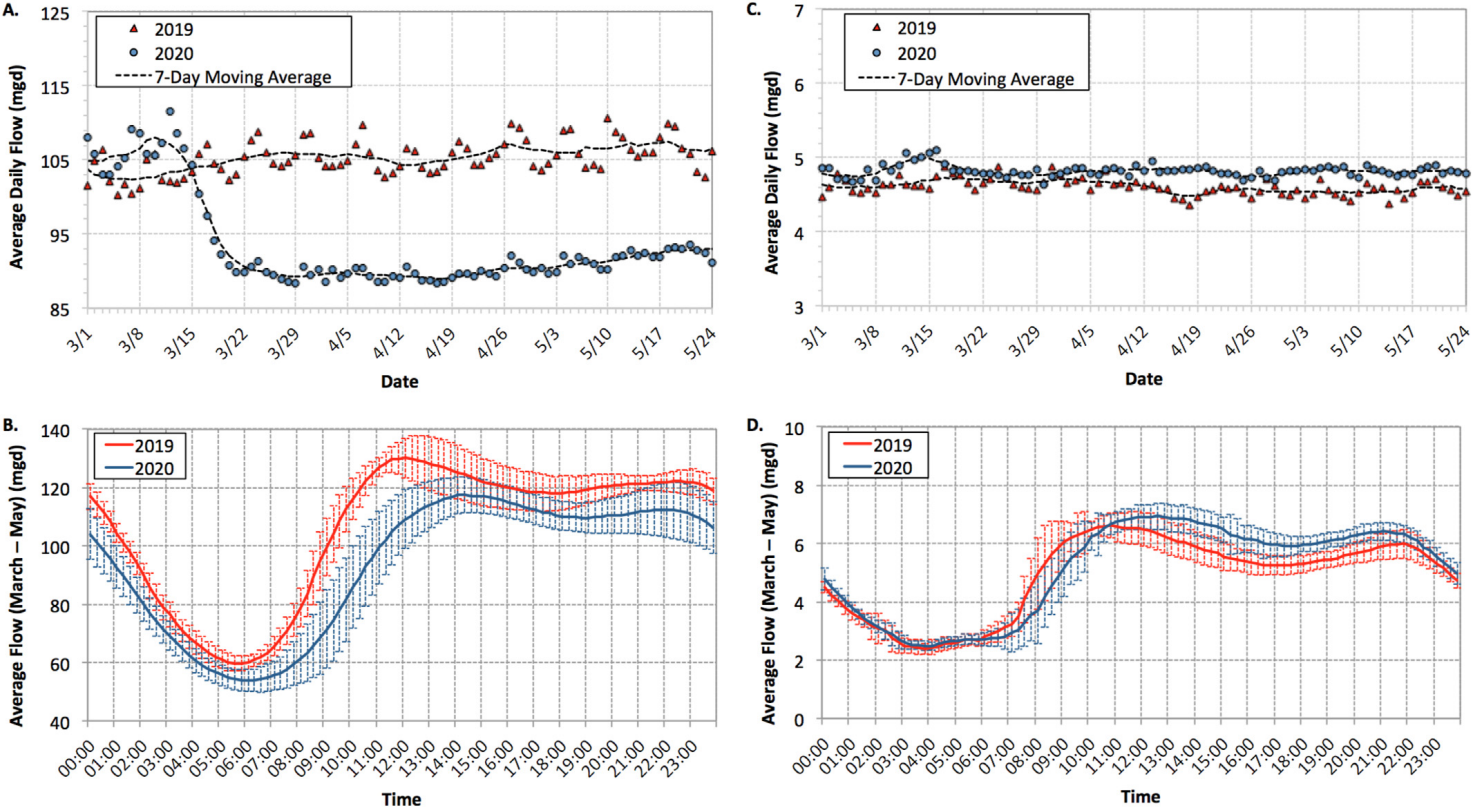
Average daily flow and diurnal flow data in million gallons per day (mgd) for (A,B) Wastewater Treatment Facility 1 and (C,D) Wastewater Treatment Facility 2. Data in B and D represent 15-min flow averages (±1 standard deviation) between early March and late May.

Fig. 3 summarizes the wastewater flow data for Facilities 1 and 2 between March and May of 2019 and 2020. Because of its service area, Facility 1 was significantly impacted by the closure of casinos and hotels along the Las Vegas Strip in mid-March. Facility 2, which serves primarily residential areas, experienced minimal impacts from COVID-19 mitigation measures, although average daily flows may have increased slightly due to people spending a greater amount of time at home. At both facilities, peak flow shifted ∼2 h later in the day when comparing 2019 versus 2020. Because the shift was observed in both facilities, with one experiencing lower flows and the other experiencing normal/higher flows, the shift was presumably due to behavioral changes rather than hydraulics. As noted earlier, the grab primary effluent samples collected between 10:00 a.m. and 11:00 a.m. correspond with the minimum observed flow rate at Facility 1, after accounting for the 5-h hydraulic retention time of the primary clarifier.

**Fig. 4:**
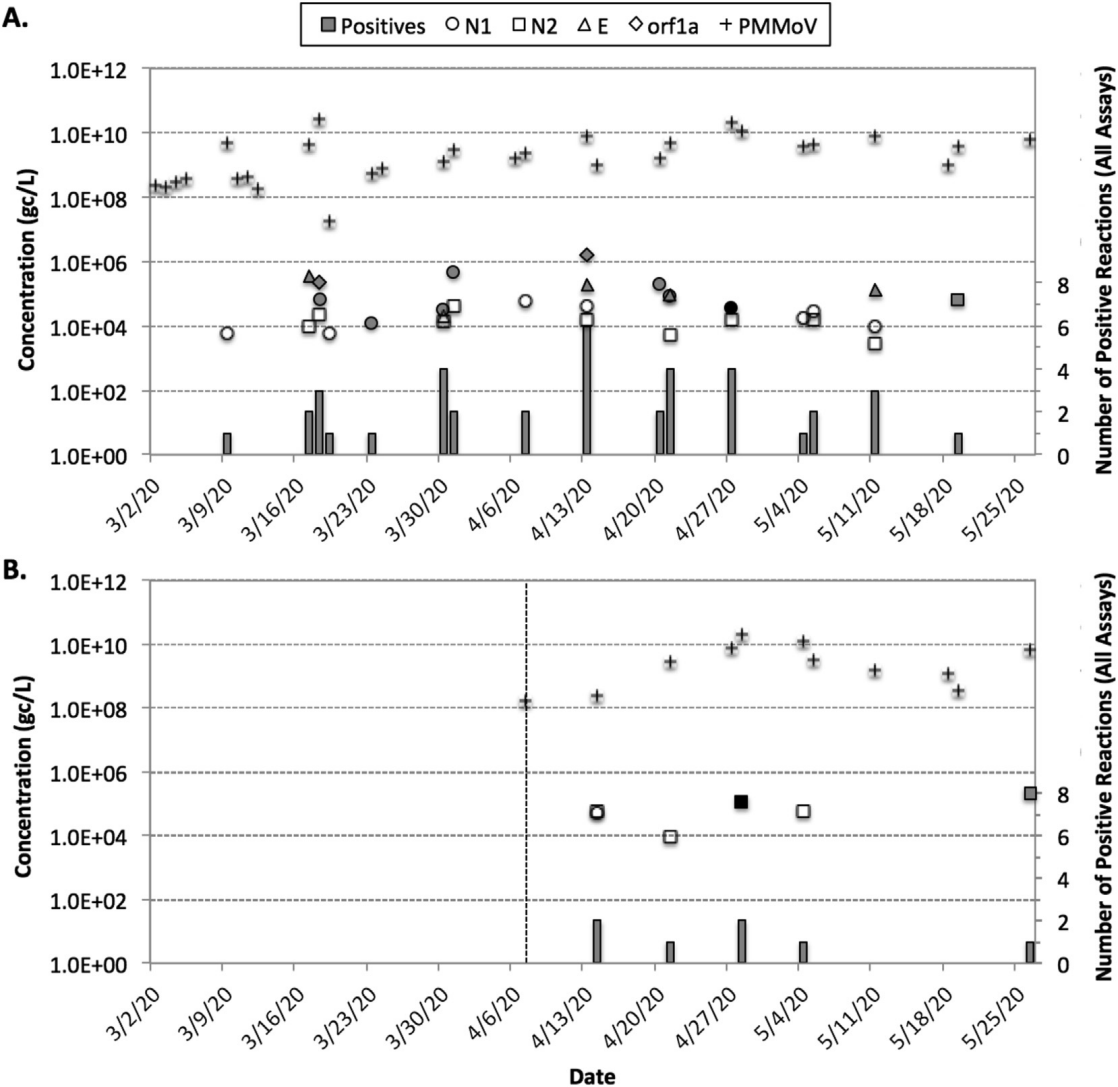
Summary of routine SARS-CoV-2 monitoring data for (A) Facility 1 from early March to late May and (B) Facility 2 from early April to late May. The symbols represent SARS-CoV-2 or PMMoV concentrations (all adjusted for equivalent sample volume and recovery). For SARS-CoV-2, open symbols indicate the sample was >LoD but < LoQ, gray symbols indicate the sample was >LoQ with detections in only one of two qPCR replicates, and black symbols indicate the sample was >LoQ with detections in both qPCR replicates. Gray columns indicate the number of positive reactions out of eight (four assays with duplicate qPCR reactions).

Fig. 4 summarizes the routine monitoring data for SARS-CoV-2 RNA. For Facilities 1 and 2, respectively, N1 was detected in 64% and 9% of all samples, N2 was detected in 48% and 45%, E_Sarbeco was detected in 20% and 0%, and *orf1a* was detected in 8% and 0%. The average Cqs for the samples with detections were 33.8 ± 1.4 for N1, 35.1 ± 1.6 for N2, 35.2 ± 1.1 for E_Sarbeco, and 33.3 ± 1.8 for *orf1a*. Similar inconsistency in assay results has been reported by Ahmed et al. (2020) in Australia, Medema et al. (2020) in the Netherlands, and Nemudryi et al. (2020) in Montana. On the other hand, Wu et al. (2020b) observed relatively consistent detections of N1, N2, and N3 in Massachusetts, and Green et al. (2020) observed consistent detections for all quantifiable samples in upstate New York. One study noted that inconsistency across assays might result from increasing prevalence of genetic variants (Penarrubia et al., 2020), but similar to Ahmed et al. (2020), many positive samples in the current study were near the LoDs and LoQs for the respective assays, which may have resulted in variability between gene targets with differing sensitivities. There were no detections during the first week of March, but there was a detection of N1 at Facility 1 on March 9th, which is consistent with the first confirmed clinical case in the local community. Both Peccia et al. (2020) and Medema et al. (2020) detected SARS-CoV-2 RNA in wastewater prior to confirmed cases in several communities.

With four SARS-CoV-2 assays and duplicate reactions for each assay, there were a total of eight qPCR reactions for each sample. Only several samples were positive for multiple assays, and many samples were positive for only one qPCR replicate for a given assay. Therefore, the number of positive reactions was used as a semi-quantitative indication of the strength of the SARS-CoV-2 genetic signal. For Facility 1, the peak signal occurred on April 13th when 4 assays and 6 of 8 total reactions were positive; several samples between March 30th and April 27th had at least 3 positive assays and 4 of 8 positive reactions. This semi-quantitative peak was somewhat consistent with the clinical peak in Fig. 2. Facility 2 never exceeded 2 positive assays and/or reactions, with the peak occurring sometime between April 13th and April 27th. Therefore, there was some temporal consistency in the data from Facilities 1 and 2, and the fact that the genetic signal was seemingly stronger at Facility 1 is consistent with the clinical data for each service area. As of May 31st, the relative prevalence in the service area for Facility 2 was 0.22% based on confirmed cases, while the relative prevalence in the immediate area surrounding Facility 1 was 0.40% on average and as high as 0.65% in one area (Fig. S3) (SNHD, 2020).

With respect to gene copy (gc) concentrations, there was only one sample from Facility 1 that was >LoQ and positive for both qPCR replicates (3.6 × 10^4^ gc/L for N1 on April 27th) and one sample from Facility 2 (1.2 × 10^5^ gc/L for N2 on April 28th). Multiple samples at Facility 1 were quantifiable based on single qPCR replicates, while only one additional sample was technically quantifiable at Facility 2. In general, concentrations at both facilities appeared to fluctuate between 10^4^ and 10^6^ gc/L. The average PMMoV concentration was approximately 4.5 × 10^9^ gc/L at both facilities, which is consistent with the literature (Symonds et al., 2018).

Liquid-phase concentrations reported in the peer-reviewed (Ahmed et al., 2020; Nemudryi et al., 2020; Wu et al., 2020b) and currently non-peer-reviewed literature (Green et al., 2020; Wurtzer et al., 2020) vary by several orders of magnitude—as low as 10^1^ gc/L in Australia to almost 10^7^ gc/L in France—but it is currently unclear whether that is due to differences in COVID-19 incidence/prevalence, sample processing methods, data interpretation (e.g., accounting for virus recovery and/or ESVs), or perhaps a combination of factors. The concentrations reported in Wu et al. (2020b) were slightly lower than the current study, although confirmed COVID-19 prevalence in their Massachusetts study area was also an order of magnitude lower at that time. That study did not appear to correct for virus recovery, and concentrations may have also been impacted by pasteurization prior to analysis. For areas with similar clinical prevalence or death rates, reported concentrations were either several orders of magnitude lower (Ahmed et al., 2020), relatively consistent (Green et al., 2020), or approximately one order of magnitude higher (Wurtzer et al., 2020) than the observed data in Southern Nevada. As noted in Ahmed et al. (2020), interlaboratory validation is a critical need for wastewater surveillance of SARS-CoV-2, and until that is addressed and more peer-reviewed studies are published, it will be difficult to reliably compare data across time and location.

### 3.3 Incidence/prevalence estimates

Approaches to estimate incidence or prevalence based on wastewater surveillance data have also varied across studies. Some studies have used a mass balance approach by coupling fecal shedding data from the literature, sewershed characteristics, and SARS-CoV-2 RNA concentrations (see Eq. (1)) (Ahmed et al., 2020; Wu et al., 2020b).

(1) $$\text{Infections }\left(\text{persons}\right)\;=\;\frac{SARSCoV2\;Concentration\;\left(gc/L\right)\;\times \;Wastewater\;Flow\;Rate\;\left(L/d\right)}{Feces\;Production\;Rate\;\left(g/person-d\right)\;\times \;Fecal\;Shedding\;Rate\;\left(gc/g\right)}$$

With this approach, it is important to capture the uncertainty and inherent variability of critical parameters, which can be accomplished to some degree with Monte Carlo simulations (Ahmed et al., 2020). Feces production rate is fairly well characterized in the literature with a median of 126–250 g/person-d, depending on location (Rose et al., 2015). Wastewater flow rate is also relatively straightforward when using average daily flow for a particular facility, although there could be artifacts of sample collection (e.g., influent vs. primary effluent and grab vs. composite) that affect the composition of the sample and how that should be considered in the model. At this time, the major source of uncertainty is the fecal shedding rate and how that varies over time, between individuals (e.g., symptomatic vs. asymptomatic), and across other demographic factors. Wölfel et al. (2020) provided an initial assessment demonstrating that fecal shedding rate varied by several orders of magnitude for a given person, with a maximum shedding rate early in the infection period and then a steady decline over as long as ∼30 days (Fig. S4). Zheng et al. (2020) reported similar viral loads in stool samples and a similar shedding duration but did not observe any clear trends over time. Assuming the Wölfel et al. (2020) trajectory, a simple mass balance approach may not be appropriate for determining overall prevalence within a community, particularly for a prolonged outbreak, but may provide a reasonable indication of *new* cases, or incidence, that might dominate the SARS-CoV-2 wastewater load.

Using the simple mass balance approach, we assumed a feces production rate of 126 g/person-d, an initial fecal shedding rate of 10^8.9^ gc/g for new cases (adapted from Wölfel et al., 2020; see Fig. S4), and average SARS-CoV-2 RNA concentrations across all assays (including estimated concentrations < LoQ) from the routine monitoring samples. These calculations resulted in estimates of ∼200 new cases per day for Facility 1 and ∼20 new cases per day for Facility 2, which equates to 0.02–0.03% daily relative incidence. Considering that confirmed daily relative incidence was 0.004% on average in April and May (Fig. 2), the wastewater estimates indicate the ascertainment ratio was approximately 5–8 in Southern Nevada during this period. This is in general agreement with Wu et al. (2020a), which suggested the ascertainment ratio was on the order of 3–20 in the United States early in the pandemic.

Green et al. (2020) used ratios of SARS-CoV-2 (i.e., infected fecal load) to crAssphage (i.e., total fecal load) as an indirect estimate of disease prevalence, while the current study focused on PMMoV to estimate incidence. SARS-CoV-2:PMMoV ratios in the current study suggested an average of ∼30 new cases per day for Facility 1 across the entire study period (i.e., 0.003% relative incidence), with an apparent peak occurring between March 23rd and April 20th. For Facility 2, the peak ratio occurred on April 14th indicating ∼15 new cases per day (i.e., 0.025% relative incidence), and the remaining data suggested fewer than 2 new cases per day (i.e., 0.003% relative incidence). These ratio-based estimates appear to align more closely with the confirmed clinical data for Southern Nevada (Fig. 2), even without adjusting for asymptomatic infections or ascertainment ratio.

**Fig. 5:**
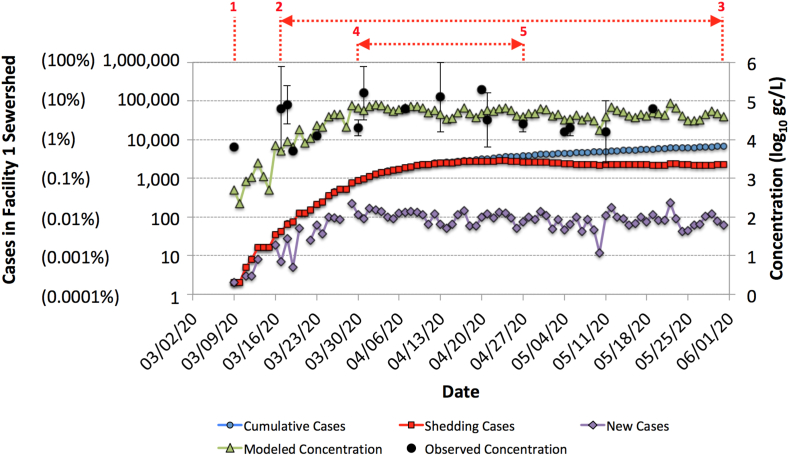
Model output (green triangles) and observed SARS-CoV-2 RNA concentrations (black circles) for the Facility 1 wastewater. The observed data represent mean log10 concentrations ±1standard deviation across all positive assays for a given sample. Purple diamonds indicate new daily cases within the sewershed [50% asymptomatic (assumed) and 50% symptomatic(clinically confirmed)], blue circles represent cumulative cases within the sewershed (asympomatic + symptomatic), and red squares indicate the number of shedding individuals at any time (modeled). The red numbers indicate days of interest: (1) first detection of SARS-CoV-2 RNA in Facility 1 wastewater, (2–3) period of consistent detections of SARS-CoV-2 RNA in wastewater, (4–5) period of strongest SARS-CoV-2 RNA signal in wastewater. Parentheses on the left vertical axis indicate relative COVID-19 incidence or prevalence within the sewershed.

Fig. 5 illustrates an alternative framework for estimating SARS-CoV-2 concentrations in Facility 1 wastewater, specifically accounting for the time-dependent shedding rate of infected individuals. In the underlying model (see Text S3), the fecal shedding rate followed the trajectory illustrated in Fig. S4 (i.e., decreasing from a maximum of 10^8.9^ gc/g to 1 gc/g over 25 days), an adaptation of Wölfel et al. (2020). The number of new cases per day was based on 50% of the clinically confirmed daily cases reported for Southern Nevada (SNHD, 2020), since Facility 1 serves approximately half of the local population, but the model also assumed an asymptomatic ratio of 50% (Day, 2020), or an ascertainment ratio of 2. An infected individual was assumed to no longer be shedding once the shedding rate dropped below 1 gc/g. The SARS-CoV-2 load from all shedding individuals on a given day was divided by the corresponding average daily flow for Facility 1.

Considering that the model predicts concentrations of 10^2^-10^3^ gc/L early in the outbreak (i.e., <LoD for this study) and that the first wastewater detection occurred during that time (i.e., March 9th), it is unlikely that the confirmed case load accurately represented COVID-19 incidence/prevalence in the community at that time. Consistent wastewater detections of SARS-CoV-2 occurred once the confirmed daily case load and cumulative cases exceeded 0.001% relative incidence and 0.01% relative prevalence, respectively. This corresponds with model concentrations of ∼10^4^ gc/L or higher, which is consistent with the observed LoDs for the SARS-CoV-2 assays. The strongest observed wastewater signal, as determined by the number of positive assays and replicate reactions (see Fig. 4), occurred when the confirmed daily case load and cumulative cases reached 0.01% relative incidence and 0.1% relative prevalence, respectively, which corresponds with a model concentration of nearly 10^5^ gc/L. The observed wastewater concentrations were generally in agreement with the model predictions, although some samples exhibited high variability between the four molecular targets.

The model assumes new cases with high shedding rates dominate the SARS-CoV-2 wastewater load, which is why the daily case load and modeled concentrations generally mirror each other in Fig. 5. Based on this shedding framework, wastewater concentrations of SARS-CoV-2 may be a useful *leading* indicator of COVID-19 when the case load is increasing by orders of magnitude over a short period of time. But due to inherent variability in SARS-CoV-2 methods, wastewater surveillance may not have sufficient resolution to detect small changes in daily case load. For much of the study period, observed and predicted wastewater concentrations fluctuated between 10^4^ and 10^5^ gc/L because daily relative incidence also fluctuated around 0.01% from late March through the end of May. Finally, the persistence of fecal shedding suggests that *clinical* data may be better suited to identify the waning portion of the COVID-19 epidemiological curve, although this could not be demonstrated in the current study because of the consistency in new daily cases through the end of May.

## 4.0 Conclusions

The COVID-19 pandemic highlighted deficiencies in rapidly deploying widespread clinical testing. This subsequently raised awareness of wastewater surveillance and its value in detecting the introduction of COVID-19 into a community, characterizing COVID-19 incidence or prevalence, and potentially informing policy measures. The scientific community rapidly developed methods to initiate wastewater surveillance of SARS-CoV-2 throughout the world, but the resulting studies often differed in terms of sample collection, processing, and/or analysis, thereby making it difficult to compare results and draw broad conclusions. This highlights the urgent need for an interlaboratory comparison of SARS-CoV-2 methodology.

Until that is completed, the current study provides guidance on certain aspects of SARS-CoV-2 wastewater surveillance. For example, SARS-CoV-2 RNA appears to show a diurnal effect in wastewater that might necessitate composite sampling. Moreover, secondary concentration methods may not be necessary and may actually be detrimental for detection and quantification of SARS-CoV-2 RNA. In any case, understanding the implications of method selection on equivalent sample volume and virus recovery is critical for accurately characterizing SARS-CoV-2 RNA concentrations. This information can be used to inform future sampling design or to retrospectively compare data from early studies.

In addition to characterizing concentrations of SARS-CoV-2 genetic markers in a specific community, the proposed model from this study highlights the potential significance of *new* cases (i.e., incidence rather than prevalence) in driving wastewater loads. The model also suggests that wastewater surveillance might be a valuable leading indicator of COVID-19 outbreaks but may be a lagging indicator for declining infection rates due to prolonged viral shedding. The value of wastewater surveillance may be partially dependent on the status of clinical testing in a given community (e.g., extent of clinical testing lags). Additional studies or meta-analyses are needed to confirm the methods and assumptions used when comparing wastewater surveillance data to infection incidence or prevalence. In particular, additional data are needed to verify the assumption in the current study of high shedding early in the infection period. Nevertheless, this study demonstrates that wastewater surveillance can provide a valuable assessment of community health conditions, particularly when clinical testing resources are stressed or uncertain.

## Data availability

All data generated or used during this study are available from the corresponding author by request.

## Declaration of competing interest

There are no conflicts to declare.

## References

[bib1] Ahmed W., Angel N., Edson J., Bibby K., Bivins A., O’Brien J.W., Choi P.M., Kitajima M., Simpson S.L., Li J., Tscharke B., Verhagen R., Smith W.J.M., Zaugg J., Dierens L., Hugenholtz P., Thomas K.V., Mueller J.F. (2020). First confirmed detection of SARS-CoV-2 in untreated wastewater in Australia: a proof of concept for the wastewater surveillance of COVID-19 in the community. Sci. Total Environ..

[bib2] Bialek S., Boundy E., Bowen V., Chow N., Cohn A., Dowling N., Ellington S., Gierke R., Hall A., MacNeil J., Patel P., Peacock G., Pilishvili T., Razzaghi H., Reed N., Ritchey M., Sauber-Schatz E. (2020). Severe outcomes among patients with coronavirus disease 2019 (COVID-19) - United States, February 12-March 16, 2020. Morb. Mortal. Wkly. Rep..

[bib3] Bibby K., Fischer R.J., Casson L.W., de Carvalho N.A., Haas C.N., Munster V.J. (2017). Disinfection of Ebola virus in sterilized municipal wastewater. PLoS Neglected Trop. Dis..

[bib4] Bivins A., North D., Ahmad A., Ahmed W., Alm E., Béen F., Bhattacharya P., Bijlsma L., Boehm A., Brown J., Buttiglieri G., Calabró V., Carducci A., Castiglioni S., Cetecioglu Z., Charkraborty S., Costa F., de los Reyes F., Delgado Vela J., Farkas K., Fernandez Cassi X., Gerba C., Gerrity D., Girones R., Gonzalez R., Haramoto E., Harris A., Holden P., Islam M.T., Jones D., Kasprzyk-Hordern B., Kitajima M., Kotlarz N., Kumar M., Kuroda K., La Rosa G., Malpei F., Matus M., McLellan S., Medema G., Meschke J.S., Mueller J., Newton R., Nilsson D., Noble R., van Nuijs A., Peccia J., Perkins A., Pickering A., Rose J., Sánchez G., Smith A., Stadler L., Stauber C., Thomas K., van der Voorn T., Wigginton K., Zhu K., Bibby K. (2020). Wastewater-based epidemiology: global collaborative to maximize contributions in the fight against COVID-19. Environ. Sci. Technol..

[bib5] Casanova L.M., Jeon S., Rutala W.A., Weber D.J., Sobsey M.D. (2010). Effects of air temperature and relative humidity on coronavirus survival on surfaces. Appl. Environ. Microbiol..

[bib6] CDC (2020). Real-time RT-PCR Panel for Detection 2019-Novel Coronavirus.

[bib7] Corman V.M., Landt O., Kaiser M., Molenkamp R., Meijer A., Chu D.K., Bleicker T., Brünink S., Schneider J., Luisa Schmidt M., GJC, Mulders D., Haagmans B.L., van der Veer B., van den Brink S., Wijsman L., Goderski G., Romette J.-L., Ellis J., Zambon M., Peiris M., Goossens H., Reusken C., Koopmans M.P., Drosten C., Victor C.M., Olfert L., Marco K., Richard M., Adam M., Daniel C.K., Tobias B., Sebastian B., Julia S., Marie Luisa S., Daphne GJC M., Bart H.L., der Veer Bas V., den Brink Sharon V., Lisa W., Gabriel G., Jean-Louis R., Joanna E., Maria Z., Malik P., Herman G., Chantal R. (2020). Detection of 2019-nCoV by RT-PCR. Euro Surveill..

[bib8] Curtis K., Keeling D., Yetka K., Larson A., Gonzalez R. (2020). Wastewater SARS-CoV-2 concentration and loading variability from grab and 24-hour composite samples. medRxiv.

[bib9] Day M. (2020). COVID-19: identifying and isolating asymptomatic people helped eliminate virus in Italian village. Br. Med. J..

[bib10] Decaro N., Elia G., Campolo M., Desario C., Mari V., Radogna A., Colaianni M.L., Cirone F., Tempesta M., Buonavoglia C. (2008). Detection of bovine coronavirus using a TaqMan-based real-time RT-PCR assay. J. Virol. Methods.

[bib11] Eisenberg J.N.S. (2020). R0: How Scientists Quantify the Intensity of an Outbreak like Coronavirus and its Pandemic Potential. https://sph.umich.edu/pursuit/2020posts/how-scientists-quantify-outbreaks.html.

[bib12] Gonzalez R., Curtis K., Bivins A., Bibby K., Weir M.H., Yetka K., Thompson H., Keeling D., Mitchell J., Gonzalez D. (2020). COVID-19 surveillance in Southeastern Virginia using wastewater-based epidemiology. Water Res..

[bib13] Green H., Wilder M., Collins M., Fenty A., Gentile K., Brittany L., Zeng T., Middleton F.A., Larsen D.A. (2020). Quantification of SARS-CoV-2 and cross-assembly phage (crAssphage) from wastewater to monitor coronavirus transmission within communities. medRxiv.

[bib14] Gundy P.M., Gerba C.P., Pepper I.L. (2009). Survival of coronaviruses in water and wastewater. Food Environ. Virol..

[bib15] Gushgari A.J., Venkatesan A.K., Chen J., Steele J.C., Halden R.U. (2019). Long-term tracking of opioid consumption in two United States cities using wastewater-based epidemiology approach. Water Res..

[bib16] Hamza I.A., Jurzik L., Überla K., Wilhelm M. (2011). Evaluation of pepper mild mottle virus, human picobirnavirus and Torque teno virus as indicators of fecal contamination in river water. Water Res..

[bib17] Haramoto E., Kitajima M., Hata A., Torrey J.R., Masago Y., Sano D., Katayama H. (2018). A review on recent progress in the detection methods and prevalence of human enteric viruses in water. Water Res..

[bib18] Hellmér M., Paxéus N., Magnius L., Enache L., Arnholm B., Johansson A., Bergström T., Norder H. (2014). Detection of pathogenic viruses in sewage provided early warnings of hepatitis A virus and norovirus outbreaks. Appl. Environ. Microbiol..

[bib19] Hill V.R., Kahler A.M., Jothikumar N., Johnson T.B., Hahn D., Cromeans T.L. (2007). Multistate evaluation of an ultrafiltration-based procedure for simultaneous recovery of enteric microbes in 100-liter tap water samples. Appl. Environ. Microbiol..

[bib20] Hovi T., Shulman L.M., Van Der Avoort H., Deshpande J., Roivainen M., De Gourville E.M. (2012). Role of environmental poliovirus surveillance in global polio eradication and beyond. Epidemiol. Infect..

[bib21] Lu R., Zhao X., Li J., Niu P., Yang B., Wu H., Wang W., Song H., Huang B., Zhu N., Bi Y., Ma X., Zhan F., Wang L., Hu T., Zhou H., Hu Z., Zhou W., Zhao L., Chen J., Meng Y., Wang J., Lin Y., Yuan J., Xie Z., Ma J., Liu W.J., Wang D., Xu W., Holmes E.C., Gao G.F., Wu G., Chen W., Shi W., Tan W. (2020). Genomic characterisation and epidemiology of 2019 novel coronavirus: implications for virus origins and receptor binding. Lancet.

[bib22] Lytle C.D., Sagripanti J. (2005). Predicted inactivation of viruses of relevance to biodefense by solar radiation. J. Virol..

[bib23] Maal-Bared R., Bastian R., Bibby K., Brisolara K., Gary L., Gerba C., Olabode L., Munakata N., Reimers R.S., Rubin A., Schaefer S., Sherchan S., Swift J. (2020). What water professionals need to know about COVID-19. Water Environ. Technol. Operations Eng..

[bib24] Medema G., Heijnen L., Elsinga G., Italiaander R., Brouwer A. (2020). Presence of SARS-Coronavirus-2 in sewage and correlation with reported COVID-19 prevalence in the early stage of the epidemic in The Netherlands. Environ. Sci. Technol. Lett..

[bib25] Miyani B., Fonoll X., Norton J., Mehrota A., Xagoraraki I. (2020). SARS-CoV-2 in detroit wastewater. J. Environ. Eng..

[bib26] Nemudryi A., Nemudraia A., Surya K., Wiegand T., Buyukyoruk M., Wilkinson R., Wiedenheft B. (2020). Temporal detection and phylogenetic assessment of SARS-CoV-2 in municipal wastewater. Cell Reports Med.

[bib27] Nishiura H., Kobayashi T., Miyama T., Suzuki A., Jung S. mok, Hayashi K., Kinoshita R., Yang Y., Yuan B., Akhmetzhanov A.R., Linton N.M. (2020). Estimation of the asymptomatic ratio of novel coronavirus infections (COVID-19). Int. J. Infect. Dis..

[bib28] Ort C., Lawrence M.G., Reungoat J., Mueller J.F. (2010). Sampling for PPCPs in wastewater systems: comparison of different sampling modes and optimization strategies. Environ. Sci. Technol..

[bib29] Ort C., Lawrence M.G., Rieckermann J., Joss A. (2010). Sampling for pharmaceuticals and personal care products (PPCPs) and illicit drugs in wastewater systems: are your conclusions valid? A critical review. Environ. Sci. Technol..

[bib30] Papp K., Moser D., Gerrity D. (2020). Viral surrogates in potable reuse applications: evaluation of a membrane bioreactor and full advanced treatment. J. Environ. Eng..

[bib31] Peccia J., Zulli A., Brackney D.E., Grubaugh N.D., Kaplan E.H., Casanovas-Massana A., Ko A.I., Malik A.A., Wang D., Wang M., Warren J.L., Weinberger D.M., Arnold W., Omer S.B. (2020). Measurement of SARS-CoV-2 RNA in wastewater tracks community infection dynamics. Nat. Biotechnol.

[bib32] Pecson B., Gerrity D., Bibby K., Drewes J.E., Gerba C., Gersberg R., Gonzalez R., Haas C.N., Hamilton K.A., Nelson K.L., Olivieri A., Rock C., Rose J., Sobsey M. (2020). Editorial Perspectives: will SARS-CoV-2 reset public health requirements in the water industry?. Environ. Sci. Water Res. Technol..

[bib33] Penarrubia A.L., Ruiz M., Porco R., Rao S.N., Juanola-Falgarona M., Manissero D., Lopez-Fontanals M., Pareja J. (2020). Multiple assays in a real-time RT-PCR SARS-CoV-2 panel can mitigate the risk of loss of sensitivity by new genomic variants during the COVID-19 outbreak. Int. J. Infect. Dis..

[bib34] Rosario K., Symonds E.M., Sinigalliano C., Stewart J., Breitbart M. (2009). Pepper mild mottle virus as an indicator of fecal pollution. Appl. Environ. Microbiol..

[bib35] Rose C., Parker A., Jefferson B., Cartmell E. (2015). The characterization of feces and urine: a review of the literature to inform advanced treatment technology. Crit. Rev. Environ. Sci. Technol..

[bib36] Sherchan S.P., Shahin S., Ward L.M., Tandukar S., Aw T.G., Schmitz B., Ahmed W., Kitajima M. (2020). First detection of SARS-CoV-2 RNA in wastewater in North America: a study in Louisiana, USA. Sci. Total Environ..

[bib37] SNHD (2020). Coronavirus Disease 2019 (COVID-19). Southern Nevada Health District. https://www.southernnevadahealthdistrict.org/coronavirus/.

[bib38] Symonds E.M., Nguyen K.H., Harwood V.J., Breitbart M. (2018). Pepper mild mottle virus: a plant pathogen with a greater purpose in (waste)water treatment development and public health management. Water Res..

[bib39] Thurston-Enriquez J.A., Haas C.N., Jacangelo J., Riley K., Gerba C.P. (2003). Inactivation of feline calicivirus and adenovirus type-40 by UV radiation. Appl. Environ. Microbiol..

[bib40] WHO (2020). Ebola Virus Disease. https://www.who.int/news-room/fact-sheets/detail/ebola-virus-disease.

[bib41] WHO (2020). Middle East Respiratory Syndrome Coronavirus (MERS-CoV) - Saudia Arabia. https://www.who.int/csr/don/05-may-2020-mers-saudi-arabia/en/.

[bib42] WHO (2003). Consensus Document on the Epidemiology of Severe Acute Respiratory Syndrome (SARS).

[bib43] Wigginton K.R., Boehm A.B. (2020). Environmental engineers and scientists have important roles to play in stemming outbreaks and pandemics caused by enveloped viruses. Environ. Sci. Technol..

[bib44] Wölfel R., Corman V.M., Guggemos W., Seilmaier M., Zange S., Müller M.A., Niemeyer D., Jones T.C., Vollmar P., Rothe C., Hoelscher M., Bleicker T., Brünink S., Schneider J., Ehmann R., Zwirglmaier K., Drosten C., Wendtner C. (2020). Virological assessment of hospitalized patients with COVID-2019. Nature.

[bib45] WRF (2020). Wastewater Surveillance of the COVID-19 Genetic Signal in Sewersheds: Recommendations from Global Experts. https://www.waterrf.org/sites/default/files/file/2020-06/COVID-19_SummitHandout-v3b.pdf.

[bib46] Wu S.L., Mertens A.N., Crider Y.S., Nguyen A., Pokpongkiat N.N., Djajadi S., Seth A., Hsiang M.S., Colford J.M., Reingold A., Arnold B.F., Hubbard A., Benjamin-Chung J. (2020). Substantial underestimation of SARS-CoV-2 infection in the United States. Nat. Commun..

[bib47] Wu F.Q., Zhang J., Xiao A., Gu X., Lee W.L., Armas F., Kauffman K., Hanage W., Matus M., Ghaeli N., Endo N., Duvallet C., Poyet M., Moniz K., Washburne A.D., Erickson T.B., Chai P.R., Thompson J., Alm E.J. (2020). SARS-CoV-2 titers in wastewater are higher than expected from clinically confirmed cases. mSystems.

[bib48] Wurtzer S., Marechal V., Jm M., Moulin L., Université S., Metis U.M.R., Atelier Z. (2020). Time course quantitative detection of SARS-CoV-2 in Parisian wastewaters correlates with COVID-19 confirmed cases. medRxiv.

[bib49] Xiao F., Tang M., Zheng X., Liu Y., Li X., Shan H. (2020). Evidence for gastrointestinal infection of SARS-CoV-2. Gastroenterol..

[bib50] Xu D., Zhang Z., Jin L., Chu F., Mao Y., Wang H., Liu M., Wang M., Zhang L., Gao G.F., Wang F.S. (2005). Persistent shedding of viable SARS-CoV in urine and stool of SARS patients during the convalescent phase. Eur. J. Clin. Microbiol. Infect. Dis..

[bib51] Ye Y., Chang P.H., Hartert J., Wigginton K.R. (2018). Reactivity of enveloped virus genome, proteins, and lipids with free chlorine and UV254. Environ. Sci. Technol..

[bib52] Ye Y., Ellenberg R.M., Graham K.E., Wigginton K.R. (2016). Survivability, partitioning, and recovery of enveloped viruses in untreated municipal wastewater. Environ. Sci. Technol..

[bib53] Zang R., Castro M.F.G., McCune B.T., Zeng Q., Rothlauf P.W., Sonnek N.M., Liu Z., Brulois K.F., Wang X., Greenberg H.B., Diamond M.S., Ciorba M.A., Whelan S.P.J., Ding S. (2020). TMPRSS2 and TMPRSS4 Mediate SARS-CoV-2 Infection of Human Small Intestinal Enterocytes.

[bib54] Zhang Y., Chen C., Zhu S., Shu C., Wang D., Song J. (2020). Isolation of 2019-nCoV from a stool specimen of a laboratory-confirmed case of the Coronavirus Disease 2019 (COVID-19). China CDC Wkly.

[bib55] Zheng S., Fan J., Yu F., Feng B., Lou B., Zou Q., Xie G., Lin S., Wang R., Yang X., Chen W., Wang Q., Zhang D., Liu Y., Gong R., Ma Z., Lu S., Xiao Y., Gu Y., Zhang J., Yao H., Xu K., Lu X., Wei G., Zhou J., Fang Q., Cai H., Qiu Y., Sheng J., Chen Y., Liang T. (2020). Viral load dynamics and disease severity in patients infected with SARS-CoV-2 in Zhejiang province, China, January-March 2020: retrospective cohort study. BMJ.

[bib56] Zhou J., Li C., Zhao G., Chu H., Wang D., Yan H.H.N., Poon V.K.M., Wen L., Wong B.H.Y., Zhao X., Chiu M.C., Yang D., Wang Y., Au-Yeung R.K.H., Chan I.H.Y., Sun S., Chan J.F.W., To K.K.W., Memish Z.A., Corman V.M., Drosten C., Hung I.F.N., Zhou Y., Leung S.Y., Yuen K.Y. (2017). Human intestinal tract serves as an alternative infection route for Middle East respiratory syndrome coronavirus. Sci. Adv..

