# Supplementary material for: Early-pandemic wastewater surveillance of SARS-CoV-2 in Southern Nevada: Methodology, occurrence, and incidence/prevalence considerations
Source: Water Res X. 2020 Dec 31;10:100086. doi: 10.1016/j.wroa.2020.100086 (PMC7774458; doi:10.1016/j.wroa.2020.100086)
Supplement: Multimedia component 1 [file mmc1.pdf]

Published in Water Research

Early-pandemic wastewater surveillance of SARS-CoV-2 in Southern Nevada: Methodology, occurrence, and incidence/prevalence considerations

Daniel Gerrity[a,b*], Katerina Papp[a], Mitchell Stoker[a], Alan Sims[a], and Wilbur Frehner[a]

[a]*Southern Nevada Water Authority, P.O. Box 99954, Las Vegas NV 89193*

[b]*Department of Civil and Environmental Engineering and Construction, University of Nevada Las Vegas, 4505 S. Maryland Parkway, Box 454015, Las Vegas, NV 89154*

*Corresponding author. Mailing address: P.O. Box 99954, MS 1970, Las Vegas, NV 89193, United States. Phone: (702) 856-3518. Email: Daniel.Gerrity@snwa.com.

Text S1. qPCR data interpretation.

cDNA synthesis. For the iScript™ Select cDNA Synthesis Kit, each reaction contained 5 µL of template, 8 µL of water, 4 µL of 5X iScript select reaction mix (1X final concentration), 2 µL of 50-µM random primer (5 µM final concentration), and 1 µL of iScript reverse transcriptase. Reactions were incubated at 25°C for 5 min, 46°C for 20 min, and 95°C for 1 min, then held at 4°C. For the Maxima First Strand cDNA Synthesis Kit, each reaction contained 5 µL of template, 9 µL of water, 4 µL of 5X Reaction mix (1X final concentration), and 2 µL of Maxima Enzyme Mix and was incubated at 25°C for 10 min, 50°C for 30 min, and 85°C for 5 min, then held at 4°C. The volume of each reaction was subsequently increased to 40 µL to accommodate multiple assays and replicates by doubling the volume of each reaction component, including increasing the template volume to 10 µL per reaction.

qPCR assays. For the two assays targeting the nucleocapsid proteins N1 and N2, duplicate reactions contained 5 µL of cDNA (as recommended by CDC), 10 µL of 2X iTaq Universal Probes Supermix (Bio-Rad Laboratories), 1.5 µL of each 6.7-µM primer/probe mix (0.5 µM final concentration) and 3.5 µL of water. For all other assays (E_Sarbeco, *orf1a*, BCoV, and PMMoV) only 1 µL of cDNA was used, and quantification was carried out in 10-µL reactions containing 5.0 µL of 2X iTaq Universal Probes Supermix or 5.0 µL of 2X iTaq Universal SYBR Green Supermix (PMMoV only), 3.5-3.7 µL of water, and variable concentrations of each primer/probe pair (Table S1). All assays were run on a CFX96 or CFX384 Touch™ Real-Time PCR Detection Systems (Bio-Rad Laboratories) set to 95°C for 30 sec for initial denaturation, followed by 44 or 49 cycles of denaturation at 95°C for 5 sec and annealing and extension at the assay-specific temperature for 30 sec (Table S1). For PMMoV,

the protocol concluded with a melt curve from 65°C to 90°C in increments of 0.5°C for 5 sec and a final plate read. Assay efficiencies were >95% for all targets.

Table S1. Summary of primer/probe concentrations and annealing temperatures for each assay.

Assay	Target Virus	Primer name	Primer/probe final concentration/reaction	Annealing Temp. (°C)	Number of qPCR cycles
N1	SARS-CoV-2	2019-nCoV_N1-F<br>2019-nCoV_N1-R<br>2019-nCoV_N1-P	0.5 µM<br>0.5 µM<br>0.5 µM	55	50
N2	SARS-CoV-2	2019-nCoV_N1-F<br>2019-nCoV_N1-R<br>2019-nCoV_N1-P	0.5 µM<br>0.5 µM<br>0.5 µM	55	50
E_Sarbeco	SARS-CoV-2	E_Sarbeco_F<br>E_Sarbeco_R<br>E_Sarbeco_P1	0.4 µM<br>0.4 µM<br>0.2 µM	58	45
orf1a	SARS-CoV-2	orf1a_F<br>orf1a_R<br>orf1a_Pb	0.2 µM<br>0.2 µM<br>0.2 µM	58	45
BCoV	Bovine Coronavirus	BCoV-F<br>BCoV-R<br>BCoV-Pb	0.4 µM<br>0.4 µM<br>0.2 µM	60	45
PMMoV (SYBR)	Pepper Mild Mottle Virus	PMMoV_F<br>PMMoV_R	0.4 µM<br>0.4 µM	55	45

The qPCR assays for E_Sarbeco and *orf1a* were originally run without probes using the iTaq Universal SYBR Green Supermix. Despite high assay efficiencies (98% for E_Sarbeco and ~90% for *orf1a*), false positive amplification was observed for both targets in many samples, prompting the inclusion of assay-specific, dual-labeled probes purchased from IDT. False positive amplification was determined by melt curve/melt peak analysis performed for each SYBR-based assay. This analysis clearly showed a significant difference between the melting temperature of the standard (all dilutions were consistent) and melting temperature of the samples (Table S2). Additionally, many samples, including some negative controls, had two uneven melt peaks with low fluorescence signals. Both instances demonstrated that amplification in samples was non-specific and unrelated to the presence of SARS-CoV-2 genetic material.

Furthermore, probe-based E_Sarbeco and *orf1a* qPCR assays did not produce amplification curves for all of the samples in question, confirming the melt curve analysis and false positive designations for the SYBR-based results.

Table S2. Summary of melting temperatures of standards and samples for SYBR-based E_Sarbeco and *orf1a* qPCR assays.

Assay	Average melting temperature of standard dilutions	Average melting temperature of samples	Range of melting temperatures for samples
E_Sarbeco	77.4 ± 0.2 °C	71.8 ± 0.9 °C	71°C - 77°C
orf1a	76.5 ± 0.2 °C	75.2 ± 4.5 °C	67°C - 81°C

qPCR standards for E_Sarbeco, orf1a, PMMoV, and BCoV. qPCR standards for E_Sarbeco, orf1a, PMMoV and BCoV assays were purchased from Integrated DNA Technologies (IDT, Skokie, IL, USA) as gBlock gene fragments. Upon receipt, they were resuspended in 1X TE buffer (10 mM Tris/0.1 mM EDTA) to 10 ng/µL according to IDT's instructions and immediately quantified with a Qubit 3.0 fluorometer and the dsDNA HS Assay kit. A stock of 10^8 gene copies (gc)/µL was made for each standard based on the quantified concentration and corresponding fragment size by diluting the purchased stock in an appropriate volume of 1X TE. Volumes of stock and buffer were determined using the ThermoFisher copy number calculator. Ten-fold serial dilutions were subsequently made from each 10^8 gc/µL stock to generate standard curves ranging from 10^8 to 10^0 gc/µL. These standard curve dilutions were frequently made anew to avoid bias caused by fragment degradation over time. To minimize inter-run variation, for example when using several qPCR plates to quantify a large number of samples for the same assay, the following adjustment was made: the raw Cq values of the standard dilution curves were compiled into a single document, and a combined regression

equation was generated by plotting their Cq values vs. the log-transformed copy number for each dilution. This combined equation was then used to re-calculate the number of copies of each target in each sample based on the sample-specific Cq value. Assay efficiency was assessed by inspecting the slope of the equation.

qPCR standards for N1 and N2. For assays targeting the nucleocapsid protein genes (e.g., N1, N2, and N3), many research groups are currently using the positive control included in the 2019-nCoV RUO Kit from IDT—a circular DNA plasmid with a listed concentration of 200,000 gc/µL. However, measured concentrations of this positive control by ddPCR have been found to be approximately 5-fold lower than the manufacturer's reported concentration (personal communication), which can lead to overestimation of SARS-CoV-2 concentration. IDT subsequently clarified that the plasmid provided in the 2019-nCoV RUO kit is technically intended as a qualitative positive control and was not intended for quantification purposes (personal communication). Additionally, circular DNA plasmids may require linearization prior to use as a qPCR standard, otherwise assay efficiency and reported concentrations may be impacted (Hou et al., 2010).

When comparing standard curves across all assays, we found substantial inconsistencies between the IDT positive control (analyzed for N1/N2 as a non-linearized plasmid and assuming the reported concentration of 200,000 gc/µL) versus the ATCC synthetic RNA standard (analyzed for N1/N2 and assuming 800,000 gc/µL) and gBlock standards (Table S3). The Cq values for high starting quantities of the IDT standard (10^6 - 10^2) were shifted 3-4 Cqs later in comparison with the same starting quantities of the ATCC standard. In contrast, Cq values for low starting quantities of the IDT standard (10^1 - 10^0) were less than the Cq values for the same concentrations of the ATCC standard, especially for the N1 target. This resulted in substantial

overestimation of the SARS-CoV-2 concentration in actual samples. Furthermore, the overestimation was not consistent across the entire range of the standard curve, rather higher Cq values (lower starting quantities or gene copies) were more affected than lower Cq values (higher starting quantities or gene copies) (Table S4). Therefore, all N1 and N2 data were ultimately quantified using the ATCC standard.

Table S3. Inter-assay comparison of quantification cycle (Cq) and corresponding starting quantities (SQ) for the SARS-COV-2 targets assessed in this study.

	Average Cq					
**Starting quantity**	**IDT N1**	**IDT N2**	**ATCC N1**	**ATCC N2**	**orf1a**	**E_Sarbeco**
1.00E+06	21.21	21.54	18.72	18.63	17.26	17.09
1.00E+05	24.82	25.26	21.69	21.78	20.36	20.36
1.00E+04	27.96	28.83	24.66	24.93	23.83	24.05
1.00E+03	30.76	31.71	27.62	28.09	27.40	27.07
1.00E+02	32.95	33.89	30.59	31.24	30.35	30.13
1.00E+01	34.31	36.24	33.56	34.39	33.36	33.03
1.00E+00	34.52	37.12	36.53	37.55	36.02	35.21
1.00E-01			39.50	40.70	35.39	

Table S4. Summary of Cq and SQ values determined by regression equations developed with the IDT and ATCC standards. These data show a general overestimation of SQ (or gene copies) by the IDT standard, and this overestimation becomes more pronounced at higher Cqs.

Sample Cq	SQ determined by regression equation from IDT standard	SQ determined by regression equation from ATCC standard	Overestimation factor
40	1.37	0.15	**9.38**
38	5.38	0.62	**8.73**
36	21.18	2.61	**8.12**
34	83.42	11.03	**7.56**
32	328.49	46.68	**7.04**
30	1293.58	197.49	**6.55**
25	39808.53	7271.46	**5.47**

Limits of detection and quantification. Limits of detection (LoDs) and limits of quantification (LoQs) were determined by quantifying sets of known test samples and statistically analyzing the data with t-tests. The known test samples included 9 replicate reactions for each dilution, with dilutions ranging from 1000 to 0.1 gc/reaction for E_Sarbeco and *orf1a* and from 8000 to 0.8 gc/reaction for N1 and N2. The analysis also included no template controls (NTCs) and blanks. The LoD for each assay was identified as the lowest dilution yielding a reliable positive signal (>50% positive reactions). To determine LoQs, a statistical analysis was performed on the lowest dilution exhibiting standard curve linearity, consistent amplification (>50% positive reactions), and valid melt curve characteristics (for the PMMoV assay). Linearity was determined through visual inspection of the standard curve obtained by plotting Cq values vs. log-transformed copy numbers. Dilutions that failed to meet these requirements were omitted from the LoQ analysis. The LoQ was then calculated using a one-sided t-test with 99% confidence (Eq. S1), as recommended by the U.S. Environmental Protection Agency.

$$\text{LoQ} = ts \qquad \text{(Eq. S1)}$$

where, t = student's t-test statistic ($\alpha = 0.01$; n-1 degrees of freedom) and s = standard deviation.

Equivalent sample volume. Equivalent sample volume (ESV) is the actual volume of original sample reflected in the reported data. The ESVs were determined by incorporating sample-specific volumes obtained at (or used after) each sample concentration (e.g., HFUF, Centricon) and molecular processing/analysis step (e.g., nucleic acid extraction, cDNA synthesis, qPCR). An example for the overall HFUF-Centricon sample concentration approach is shown in Eq. S2. Sample concentrations were calculated by dividing the gene copy (gc) number obtained for each assay (gc/reaction) by the corresponding ESV.

$$\mathrm{ESV\ (mL)} = (0.005/0.04) \times (0.01/0.06) \times (0.35/1.0) \times (45/200) \times 10{,}000 = 16.4\ \mathrm{mL} \quad \text{(Eq. S2)}$$

Numerators (top labels, left to right): Volume of cDNA template for N1/N2 assays; Volume of extract used to make cDNA; Volume of Centricon pellet for extraction; Volume through Centricon (varies); Initial 10-L Sample Volume.

Denominators (bottom labels, left to right): Total volume of cDNA template; Total volume of nucleic acid extract; Total volume of Centricon pellet (varies); Total volume of HFUF concentrate (varies).

Virus recovery. Virus recovery was determined by (a) spiked vaccine-strain bovine coronavirus (BCoV) and (b) ambient levels of pepper mild mottle virus (PMMoV). The BCoV stock (Calf-Guard, Zoetis, Parsippany, NJ, USA) was obtained from Mark Borchardt and Susan Spencer at the U.S. Department of Agriculture. The lyophilized material was resuspended in 3 mL of 1X TE buffer (stock solution) and then diluted 4.35-fold in 1X TE buffer (diluted stock solution). The diluted stock solution was titered via direct extraction (350-µL volume) using the Purelink Viral RNA/DNA Mini Kit (ThermoFisher Scientific, Waltham, MA) and then quantification by RT-qPCR as described earlier. After correcting for dilution, the concentration of the 3-mL resuspended stock solution was determined to be 1.6×10^{8} gc/mL, and the concentration of the diluted spiking stock was determined to be 3.6×10^{7} gc/mL. The baseline concentrations used in PMMoV recovery calculations were based on direct extraction of wastewater samples prior to sample concentration.

Recovery experiments evaluated recovery for the overall sample concentration methods and also isolated each step in the process (when secondary concentration was employed). All recovery experiments involving HFUF were carried out by spiking 1 mL of the diluted BCoV stock (3.6×10^{7} gc/**mL**) into 10 L of wastewater (final concentration of ~3.6×10^{6} gc/**L**). For recovery experiments intending to isolate small-volume concentration (i.e., Centricon and PEG),

experiments were carried out by spiking 150-250 μL of the diluted BCoV stock (3.6×10^7 gc/**mL**) into 150-250 mL of wastewater (final concentration of 3.6×10^7 gc/**L**).

False positives with E_Sarbeco qPCR assay. To examine potential cross-reactivity between BCoV and SARS-CoV-2, the diluted BCoV stock solution was assayed on 4 occasions for the N1, N2, E_Sarbeco, and *orf1a* genes (in duplicate)—a total of 32 qPCR reactions. The N1/N2 qPCR assays were carried out in 20-μL reaction volumes, while 10-μL reaction volumes were used for E_Sarbeco and *orf1a*. The SARS-CoV-2 assays did not cross-react with BCoV, except for one BCoV extract showing a positive signal in duplicate reactions for the E_Sarbeco assay (Cq = 34.90±0.40 and starting quantity = 3.97±1.06 gc/reaction). The E_Sarbeco assay also yielded a positive signal in 22% of negative controls, with Cqs ranging from 34.00 to 36.30 (corresponding starting quantities of 7.22 and 1.71 gc/reaction). The other assays showed no amplification in all negative controls and negative reverse transcription (RT) controls. Other researchers have also observed false positive amplification in negative controls with the E_Sarbeco assay (Konrad et al., 2020; personal communication).

Negative controls. Negative extraction controls were included in each RNA/DNA extraction, negative RT controls were included in each cDNA synthesis step, and no template controls were included in each qPCR run as part of the standard curve. No amplification was observed in the negative controls, with the exception of the E_Sarbeco assay described above.

Chloroform-butanol extraction following PEG precipitation. Early experiments with two separate primary effluent samples compared HFUF followed by Centricon centrifugal ultrafiltration, PEG precipitation, and PEG precipitation supplemented with chloroform-butanol extraction. Based on the Cq and ESV summary below, chloroform-butanol extraction was omitted from future PEG experiments. Virus recovery was not evaluated for these samples.

Table S5. Cq and ESV summary for Centricon, PEG, and PEG+extraction evaluation.

Assay	Centricon		PEG		PEG + Chloroform-Butanol	
	Sample 1	Sample 2	Sample 1	Sample 2	Sample 1	Sample 2
ESV[1]	6.2 mL	42 mL	74 mL	46 mL	12 mL	7.3 mL
N1	35.8 (1/2)	32.6 (1/2)	32.6 (1/2)	32.0 (1/2)	ND	ND
N2	38.6 (1/2)	34.4 (1/2)	ND	35.2 (1/2)	ND	ND
E_Sarbeco	ND	36.6 (2/2)	ND	ND	ND	ND
orf1a	ND	ND	33.3 (1/2)	38.3 (1/2)	ND	ND

[1]ESV based on 5 uL of template cDNA; [2]Parentheses indicate number of positive qPCR reactions for each assay

Text S2. Hydraulic considerations for wastewater surveillance of SARS-CoV-2.

The top-left frame of Figure S1 illustrates the hypothetical concentration profile of a ubiquitous, conservative indicator of wastewater influence and/or fecal contamination, such as sucralose or pepper mild mottle virus (PMMoV). For these constituents, the corresponding wastewater signal may be relatively constant throughout the day, with only a small influence from diurnal cycles. These indicators can be used as internal controls for a general assessment of method performance, and fecal indicators such as PMMoV can potentially be used to indicate the 'total' fecal load represented in a particular wastewater sample.

The remaining frames in Figure S1 illustrate hypothetical concentration profiles for the genetic signal of SARS-CoV-2 in the primary clarifier effluent at a wastewater treatment facility. The primary clarifier was assumed to have a hydraulic retention time of 5 hours. The concentration profile is based on a single toilet flush at 6:00 am from a COVID-19-positive individual shedding $10^{8.9}$ gene copies (gc) of SARS-CoV-2 per gram of feces. The toilet flush was assumed to contain 126 grams of feces in 10 L of water, with the water entering the sewer system over the course of 1 minute. The 1-min toilet flush was assumed to mix completely with the instantaneous flow of sewage (1 million people generating 350 liters/person-day = 2.4×10^5 L/min). This results in an equivalent SARS-CoV-2 concentration of $\sim4\times10^5$ L/min in a single 'plug' of sewage entering a wastewater treatment facility, assuming ideal plug flow through the sewer.

The bottom-left frame illustrates the hypothetical SARS-CoV-2 concentration profile in the primary clarifier effluent assuming plug flow conditions through the primary clarifier. In this scenario, the entire SARS-CoV-2 load exits the primary clarifier at 11:00 am with no dilution or dispersion effects (i.e., instantaneous signal spike of 4×10^5 gc/L). The top-right frame illustrates

the corresponding concentration profile assuming a non-ideal reactor with dispersion (baffle factor = 0.7). In this scenario the peak signal at 11:00 am is diluted by a factor of ~240, but the SARS-CoV-2 load spread out over ~8 hours, albeit with even greater dilution ratios. Finally, the bottom-right frame assumes an ideal completely stirred tank reactor (CSTR). In this scenario, the signal is immediately diluted by a factor of ~20 due to complete mixing with the contents of the CSTR, and the signal declines steadily over many hours.

Figure S1. Hydraulic considerations for wastewater surveillance of SARS-CoV-2.

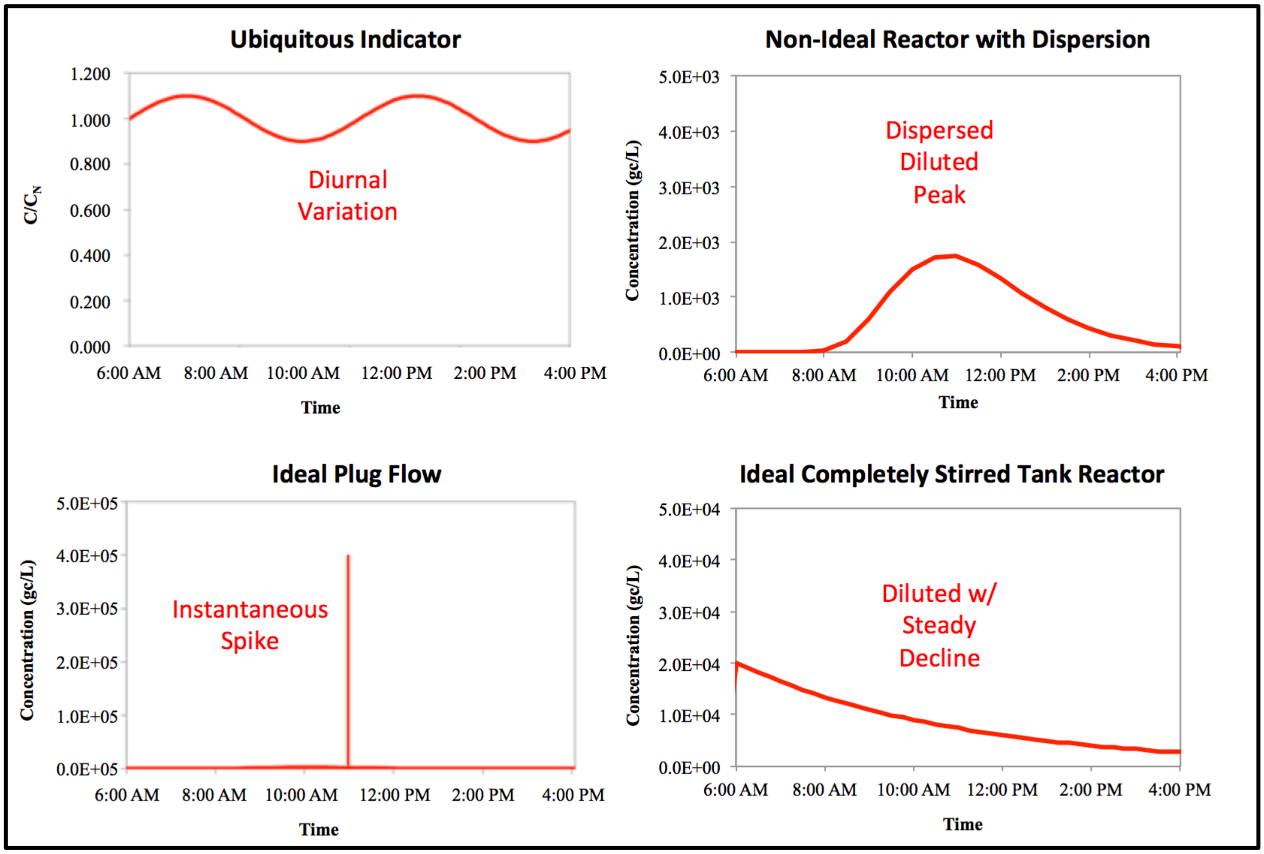

Table S6. Relative increase in SARS-CoV-2 concentration (i.e., X-fold) in composite samples compared to corresponding grab primary effluent samples. All concentrations were adjusted for equivalent sample volume and virus recovery, and calculations were based on geometric mean concentrations across all SARS-CoV-2 assays for a given sample. At least one replicate was >LoQ for all samples indicating an X-fold increase. For the 3/2/20 and 3/9/20 samples, the grab primary effluent, composite influent, and composite primary effluent samples were all concentrated by Centricon only. For the remaining samples, the grab primary effluent samples were concentrated by HFUF-Centricon (2.1% recovery for SARS-CoV-2 and 0.12% recovery for pepper mild mottle virus), and the composite samples were concentrated by Centricon only (55% recovery for SARS-CoV-2 and 1.3% recovery for pepper mild mottle virus).

**Sample Date**	**SARS-CoV-2**		**Pepper Mild Mottle Virus (PMMoV)**	
	**Comp. Influent**	**Comp. Primary**	**Comp. Influent**	**Comp. Primary**
3/2/20	ND	ND	534	596
3/9/20	N/A[1]	NS	28	NS
3/18/20	4	4	2	4
3/23/20	57	NS	403	NS
3/30/20	ND	9	17	101
3/31/20	5	NS	60	NS
4/6/20	N/A[2]	NS	19	NS
4/13/20	2	NS	16	NS
4/20/20	2	NS	78	NS
4/21/20	NS	11	NS	16
4/27/20	3	NS	53	NS
**Minimum**	**2X Higher**	**4X Higher**	**2X Higher**	**4X Higher**
**Maximum**	**57X Higher**	**11X Higher**	**534X Higher**	**596X Higher**
**Average**	**12X Higher**	**8X Higher**	**121X Higher**	**179X Higher**

ND = Non-detect for all assays; NS = Composite not sampled; [1]N1 Detected in grab primary effluent only; [2]N2 detected in composite influent only.

Figure S2. Supplementary COVID-19 public health data (SNHD, 2020; Nevada, 2020).

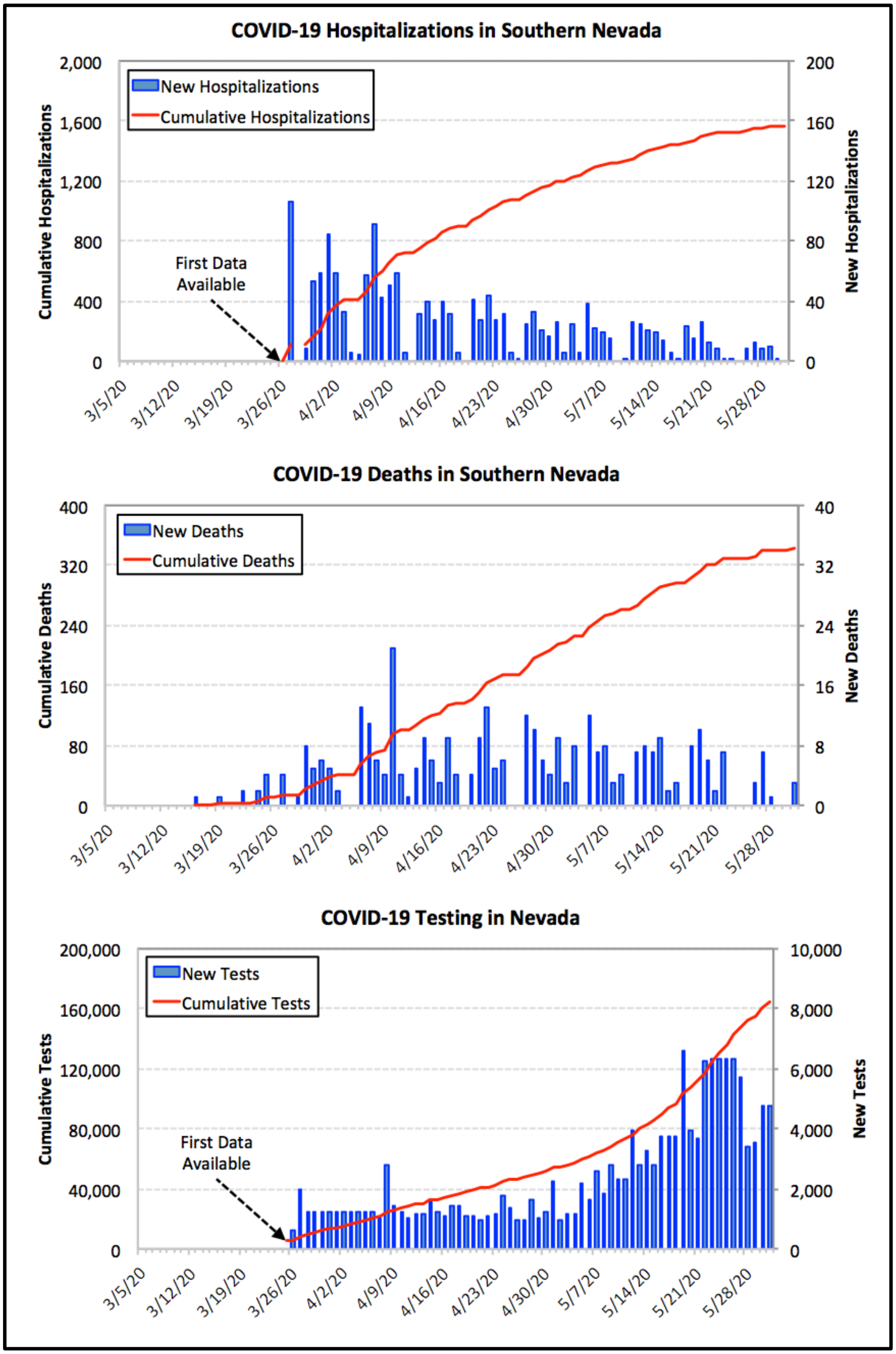

Figure S3. Relative prevalence of COVID-19 (cases per 100,000 people) based on confirmed case data as of May 2020 (SNHD, 2020). Service areas for Facilities 1 and 2 are not shown on the map to maintain their anonymity.

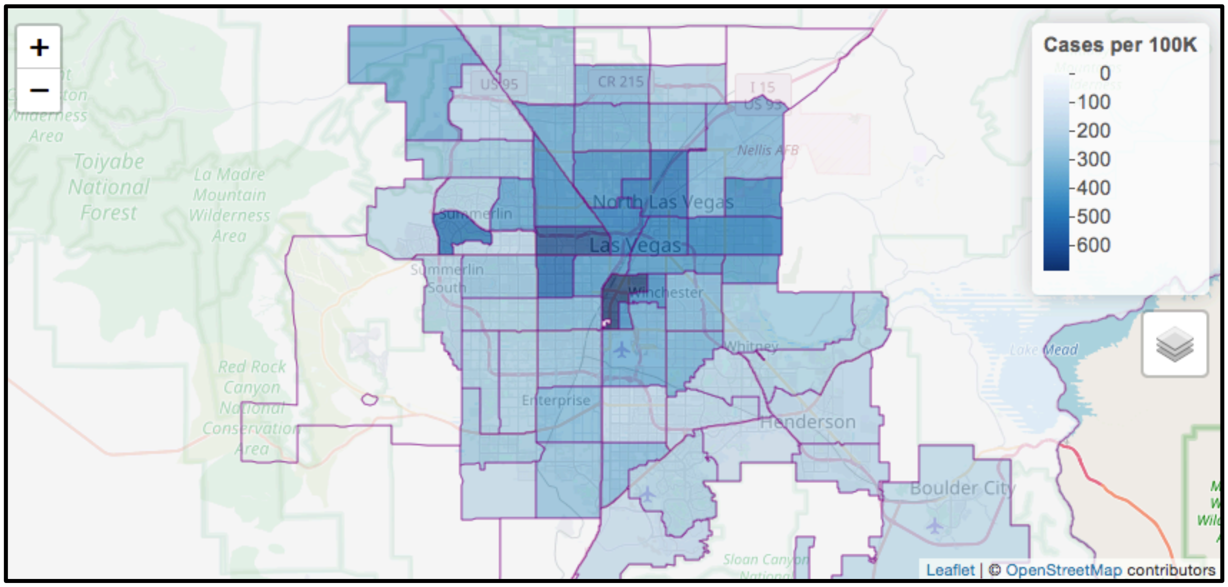

Figure S4. Fecal shedding data from Wolfel et al. (2020). The data indicated by colored lines (blue, green, red) were assumed to represent the most likely shedding trajectory for infected individuals and were used to develop the regression equation. This assumption was supported by comparing observed data with model output for Southern Nevada wastewater.

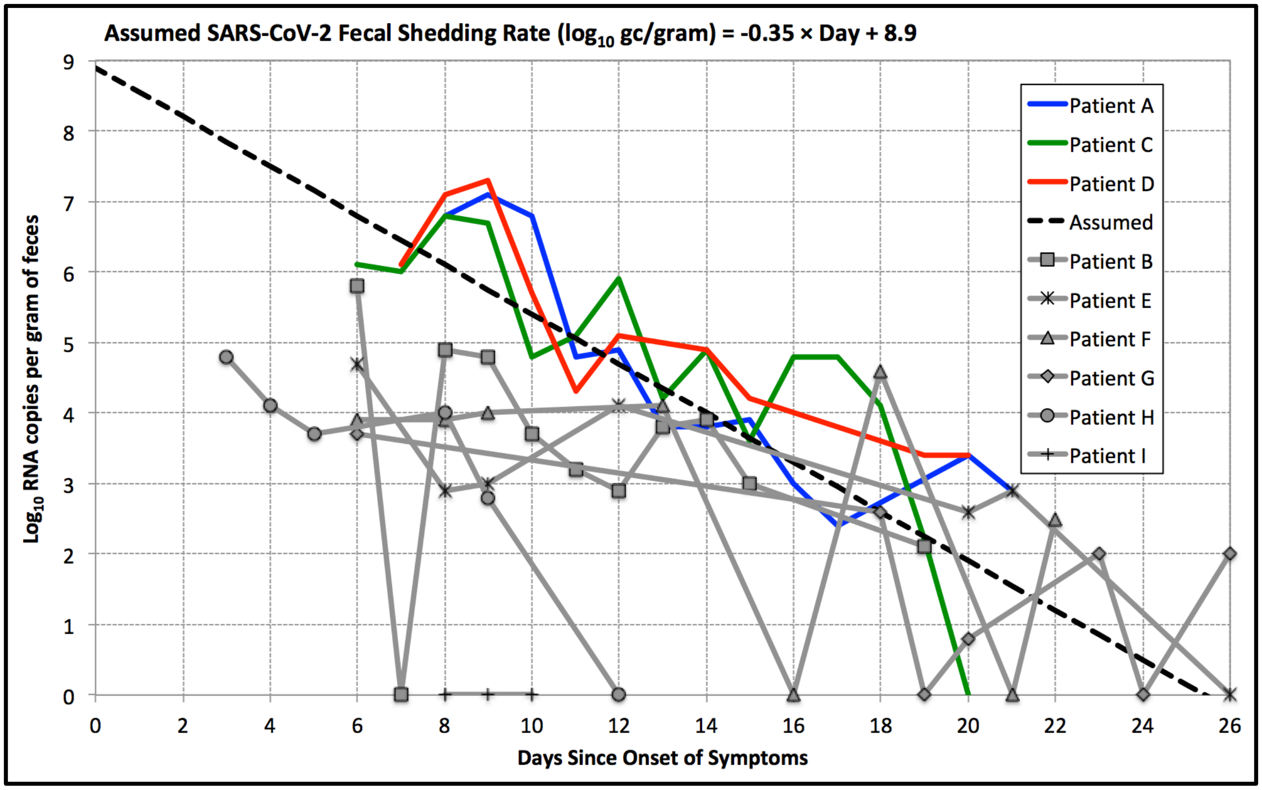

Text S3. MATLAB code for SARS-CoV-2 wastewater model.

The following script can be executed in MATLAB (MathWorks, Natick, MA, USA) to develop a predicted SARS-CoV-2 wastewater concentration based on sewershed characteristics. All portions of the code in bold font can be adapted to the sewershed in question and modified to user specifications as additional data become available (e.g., viral shedding distributions from clinical research). Sewershed-specific COVID-19 case data should accompany the code as a .csv file with two columns: column 1 = duration of outbreak (i.e., number of days) and column 2 = ***cumulative*** confirmed case count. The .csv file should include cumulative case data from the beginning of the pandemic (i.e., cases should start at 0). If not, the user may have to assume values for the 'left-censored' case data or disregard the initial model output because of how the code is structured. Finally, this code assumes a constant average daily wastewater flow rate (based on per capita wastewater generation rate multiplied by sewershed population) but can be modified if desired by the user to account for fluctuations over time.

```
clear
clc
tic

%% User Inputs
population = 1021124; % population of sewershed, persons
minfeces = 126; % min for uniform distribution, grams of feces/person-day
maxfeces = 126; % max for uniform distribution, grams of feces/person-day
wwgeneration = 365; %per capita wastewater generation rate, L of WW/person-day
initialshedding = 8.9; % initial viral load in feces, log10gc/g
sheddingdecay = 0.35; % daily decrease in virus shedding rate, log10gc/g per day
asymptomatic = 0.5; % asymptomatic ratio

%% Read files
rawcasedata=csvread('cases.csv',0,1); % 1st column = day and 2nd column = cumulative cases in sewershed
duration = length(rawcasedata); % duration of outbreak, days
casedata = zeros(duration,1); % only reads 2nd column of case file with no headers
for i = 1:duration
   casedata(i,1) = round(rawcasedata(i,1)/(1-asymptomatic));
end

%% Calcs
```

```
ww = population*wwgeneration; % wastewater flow rate, L/day
wwmgd = ww/3.785/1000000; % wastewater flow rate, mgd
sheddingarray = zeros(population,duration+1); % empty array for shedding rate
activearray = zeros(population,duration+1); % empty array for active shedding accounting
concarray = zeros(population,duration+1); % empty array for active shedding accounting
totconc = zeros(1,duration+1);
activeshedders = zeros(1,duration+1);
case_change = zeros(1,duration+1);
case_previous = 0;

for i = 1:duration % i = day of outbreak
   case_change(1,i+1) = casedata(i,1)-case_previous;
   start = case_previous + 1;
   finish = casedata(i,1);

      for j = 1:population
         sheddingarray(j,i+1)=sheddingarray(j,i)-sheddingdecay;
      end

      for j = start:finish
         sheddingarray(j,i+1)=initialshedding;
      end

   case_previous = casedata(i,1);
end

for i = 1:duration % i = day of outbreak
      for j = 1:population
         if sheddingarray(j,i+1)>0
            activearray(j,i+1) = 1;
         else
            activearray(j,i+1) = 0;
         end
      end
end

for i = 1:duration % i = day of outbreak
      for j = 1:population
         concarray(j,i+1)=activearray(j,i+1)*randi([minfeces,maxfeces],1)*(10^sheddingarray(j,i+1))/ww;
      end
end

for i = 1:duration
   totconc(1,i+1)=sum(concarray(:,i+1));
end

for i = 1:duration
   activeshedders(1,i+1)=sum(activearray(:,i+1));
end

confirmed_cases = rawcasedata;
total_cases = confirmed_cases/asymptomatic;
totconc=transpose(totconc);
wwmgd % outputs wastewater flow rate in mgd as a user check
totconc % outputs predicted wastewater concentration by day in gc/L
toc
```

References

Hou, Y., Zhang, H., Miranda, L., Lin, S., 2010. Serious overestimation in quantitative PCR by circular (supercoiled) plasmid standard: Microalgal *pcna* as the model gene. PLoS One 5(3): e9545.

Konrad, R., Eberle, U., Dangel, A., Treis, B., Berger, A., Bengs, K., Fingerle, V., Liebl, B., Ackermann, N., Sing, A., 2020. Rapid establishment of laboratory diagnostics for the novel coronavirus SARS-CoV-2 in Bavaria, Germany, February 2020. Eurosurveillance 25(9): pii=2000173.

Nevada, 2020. Daily Situation Reports 2020. Nevada Governor Steve Sisolak. http://gov.nv.gov/News/DailySituationReports/Daily_Situation_Reports/. Accessed: June 19, 2020.

SNHD, 2020. Coronavirus Disease 2019 (COVID-19). Southern Nevada Health District. https://www.southernnevadahealthdistrict.org/coronavirus/. Accessed: June 19, 2020.

Wölfel, R., Corman, V.M., Guggemos, W., Seilmaier, M., Zange, S., Müller, M.A., Niemeyer, D., Jones, T.C., Vollmar, P., Rothe, C., Hoelscher, M., Bleicker, T., Brünink, S., Schneider, J., Ehmann, R., Zwirglmaier, K., Drosten, C., Wendtner, C., 2020. Virological assessment of hospitalized patients with COVID-2019. Nature 581.
